# CCL20 triggered by chemotherapy hinders the therapeutic efficacy of breast cancer

**DOI:** 10.1371/journal.pbio.2005869

**Published:** 2018-07-27

**Authors:** Weilong Chen, Yuanyuan Qin, Dong Wang, Lei Zhou, Yin Liu, Sheng Chen, Liang Yin, Yaoxing Xiao, Xiao-Hong Yao, Xiaoli Yang, Wei Ma, Weifeng Chen, Xueyan He, Lixing Zhang, Qifeng Yang, Xiuwu Bian, Zhi-ming Shao, Suling Liu

**Affiliations:** 1 School of Life Science, The CAS Key Laboratory of Innate Immunity and Chronic Disease, University of Science & Technology of China, Hefei, Anhui, China; 2 Fudan University Shanghai Cancer Center & Institutes of Biomedical Sciences, Shanghai Medical College, Key Laboratory of Breast Cancer in Shanghai, Innovation Center for Cell Signaling Network, Cancer Institutes, Fudan University, Shanghai, China; 3 Department of Oncology, Department of Breast Surgery, Shanghai Medical College, Fudan University, Shanghai, China; 4 The department of breast surgery, The First People's Hospital of Zhenjiang City, Zhenjiang, China; 5 Department of Pathology, Fudan University Shanghai Cancer Center, Shanghai, China; 6 Institute of Pathology and Southwest Cancer Center, Southwest Hospital, Third Military Medical University, and Key Laboratory of Tumor Immunopathology, Ministry of Education of China, Chongqing, China; 7 National Engineering Research Center for Functional Food, School of Food Science and Technology, Jiangnan University, Wuxi, China; 8 Department of Breast Surgery, Qilu Hospital, Shandong University, China; Terry Fox Laboratory, Canada

## Abstract

Chemotherapeutic resistance in triple-negative breast cancer (TNBC) has brought great challenges to the improvement of patient survival. The mechanisms of taxane chemoresistance in TNBC have not been well investigated. Our results illustrated C-C motif chemokine ligand 20 (CCL20) was significantly elevated during taxane-containing chemotherapy in breast cancer patients with nonpathologic complete response. Furthermore, CCL20 promoted the self-renewal and maintenance of breast cancer stem cells (BCSCs) or breast cancer stem-like cells through protein kinase Cζ (PKCζ) or p38 mitogen-activated protein kinase (MAPK)-mediated activation of p65 nuclear factor kappa B (NF-κB) pathway, significantly increasing the frequency and taxane resistance of BCSCs. Moreover, CCL20-promoted NF-κB activation increased ATP-binding cassette subfamily B member 1 (ABCB1)/multidrug resistance 1 (MDR1) expression, leading to the extracellular efflux of taxane. These results suggested that chemotherapy-induced CCL20 mediated chemoresistance via up-regulating ABCB1. In addition, NF-κB activation increased CCL20 expression, forming a positive feedback loop between NF-κB and CCL20 pathways, which provides sustained impetus for chemoresistance in breast cancer cells. Our results suggest that CCL20 can be a novel predictive marker for taxane response, and the blockade of CCL20 or its downstream pathway might reverse the taxane resistance in breast cancer patients.

## Author summary

Breast cancers are divided into several different subtypes according to the expression of three receptors: estrogen receptor (ER), progesterone receptor (PR), and human epidermal receptor 2 (HER2). One subtype of breast cancer is known as triple-negative breast cancer (TNBC) because of lack of expression of all three receptors. TNBC is highly malignant and susceptible to developing drug resistance during chemotherapy. However, the reasons for resistance have not been well explained. We found that a secretory protein called C-C motif chemokine ligand 20 (CCL20) responds to taxane chemotherapy in TNBC and promotes the expression of a drug efflux pump through a specific signaling pathway (CCL20-protein kinase Cζ [PKCζ]/p38 mitogen-activated protein kinase [MAPK]-nuclear factor kappa B [NF-κB]-ATP-binding cassette subfamily B member 1 [ABCB1]). Increasing levels of CCL20 result in expanding breast cancer stem-like cell population and chemotherapeutic resistance in breast cancer cells. Breast cancer stem-like cells are a special population of cells with self-renewal and differentiation potential within the tumor, which are often the culprit of tumor reoccurrence, drug resistance, and metastasis. Our results suggest that blocking CCL20 or its downstream signal can significantly improve the therapeutic efficacy of TNBC.

## Introduction

Breast cancer is one of the most common cancers diagnosed among women, accounting for nearly 1 in 3 cancers [1]. The recurrence, metastasis, and drug resistance in the course of chemotherapy have brought great threat to breast cancer patients [2,3], especially as chemoresistance limits the effectiveness of chemotherapeutic agents to a large extent.

Although the triple-negative breast cancers (TNBCs) are generally very susceptible to chemotherapy initially because of the highly proliferative capacity, early complete response (CR) does not correlate with overall survival (OS) [4]. Chemoresistance emerges during therapy because tumor cells are able to maintain viability following chemotherapeutic exposure via undergoing alternative cellular fates such as autophagy, cellular senescence, and therapeutic induced senescence (TIS), although cytotoxic chemotherapy aims to kill cancer cells through apoptosis [5]. There are several specific ways to regulate chemoresistance of TNBC. Regulation of ATP-binding cassette (ABC) transporters and β-tubulin III subunit, disorder in enzymes critical in DNA replication and repair, alterations in genes involved in apoptosis, and drug inactivation/detoxification and abnormal regulation of key signaling pathways such as aberrant activation of nuclear factor kappa B (NF-κB) or AKT activity all participate in chemoresistance in TNBC [6]. These regulatory mechanisms can help tumor cells escape apoptosis induced by chemotherapeutic drugs to acquire the ability of chemoresistance in TNBCs. Besides, more and more studies showed that breast cancer stem cells (BCSCs), which represent a distinct population that can be prospectively isolated from the total tumor cell population and have clonal long-term repopulation and self-renewal capacity, are responsible for chemotherapy resistance [7]. The differential and diverse regulation of BCSCs also influences the chemoresistance of TNBCs.

In addition to intrinsic genetic changes in cells as described above, recent data suggest that chemoresistance can also result from cell-extrinsic factors such as growth factors and cytokines [8]. Both malignant and cancer-associated normal cells in the tumor niche affect the balance of cytokines that play pivotal roles in regulating tumor cell proliferation, survival, and chemoresistance [9–11]. Gilbert and Hemann’s study in lymphoma model described an interleukin 6 (IL-6)-triggered cytokine-chain response that can promote therapeutic resistance to genotoxic chemotherapy of lymphoma cells [10]. In addition to IL-6, some other cytokines are also able to regulate tumor progression and chemotherapeutic resistance in an autocrine manner in multiple cancers [12]. Although both the extrinsic and intrinsic factors have revealed the possible clues to chemotherapeutic resistance in TNBC, there are still some critical questions to be answered in clinical practice. During the taxane (taxol or docetaxel) therapies for TNBC patients, the risk factors for determining drug resistance and the underlying mechanisms remain unknown. And there is a lack of effective markers to indicate the chemoresistance of TNBC. Through studies analyzing breast cancer patients’ serum, tumor tissue sample, survival prognosis, and pathologic response to taxane, we identified a chemokine, C-C motif chemokine ligand 20 (CCL20), that might predict the risk of taxane resistance in TNBC patients. Moreover, our studies in both TNBC cell line–derived xenograft (CDX) and patient-derived xenograft (PDX) mouse models showed that the blockade to CCL20 or CCL20-mediated downstream molecules could attenuate BCSCs and reverse chemoresistance and significantly improved the chemotherapeutic efficacy of taxane to TNBC.

CCL20, a member of the C-C motif chemokine subfamily, participates in many diseases’ progression—including rheumatoid arthritis (RA), psoriasis, and immune response—and promotes the malignancy in colorectal cancer and lung cancer [13,14]. However, the role of CCL20 in cancers has not been well elucidated. More importantly, little work has been done on the role of CCL20 in regulating the tumor progression and chemoresistance in breast cancer, especially TNBC, and the regulation mechanisms still remain unknown. Our current studies showed that taxane-induced CCL20 could promote self-renewal of BCSCs through the activation of p65 NF-κB, which was mediated by protein kinase Cζ (PKCζ) and p38 mitogen-activated protein kinase (MAPK) activity. The activation of p65 NF-κB further promoted transcription of both ATP-binding cassette subfamily B member 1 (ABCB1) and CCL20, which sequentially increased extracellular efflux of taxane and enhanced CCL20 effect via a positive feedback loop, leading to the taxane-resistance of TNBCs and promoting breast cancer progression with worse prognosis.

## Results

### CCL20 elevation was closely correlated with the nonpathologic complete response (non-pCR) in breast cancer patients receiving taxane-containing neoadjuvant chemotherapy (NAC)

**Fig 1:**
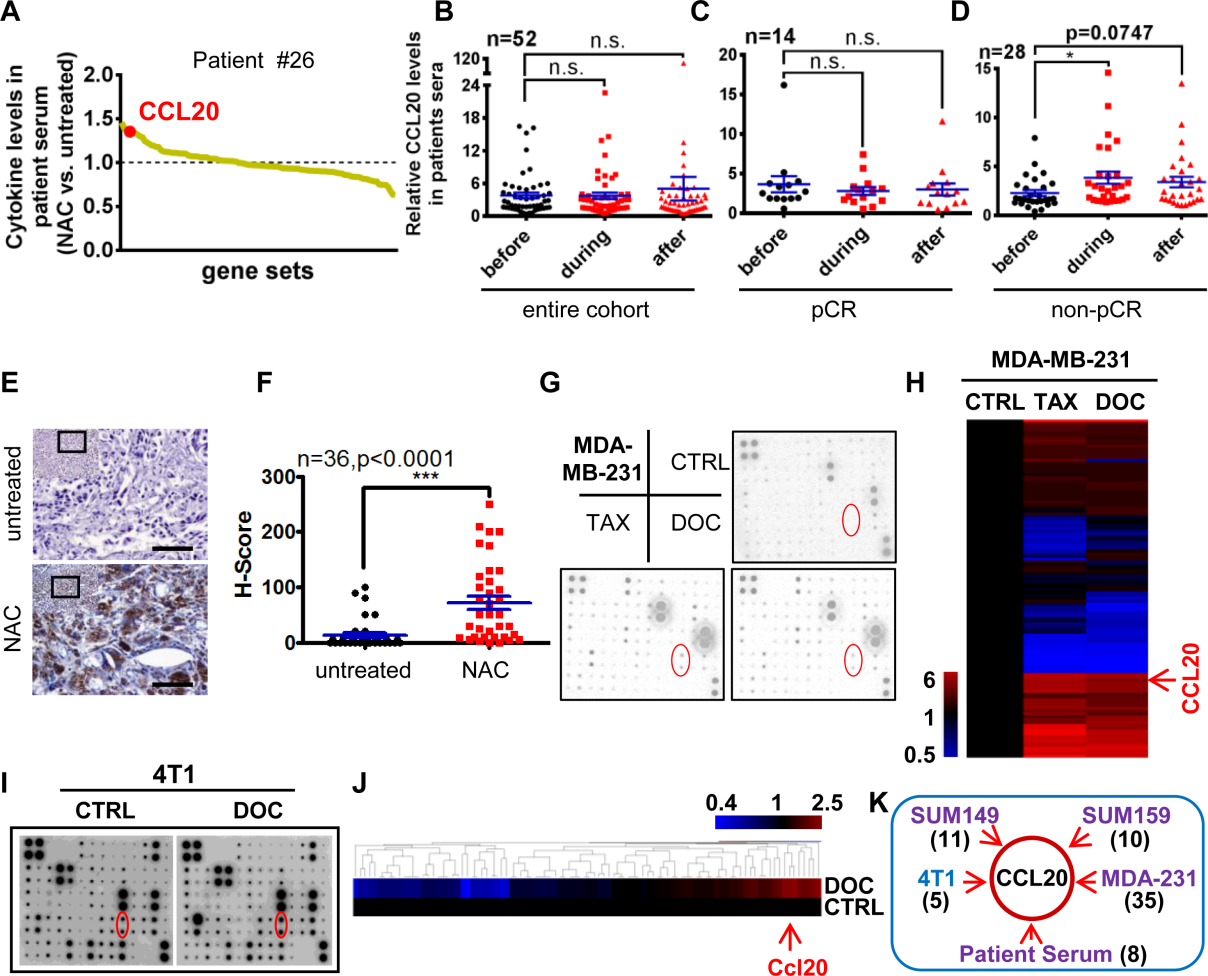
CCL20 elevation was closely correlated with the non-pCR in breast cancer patients receiving taxane-containing NAC. (A) Cytokine antibody array was carried out with sera before NAC (untreated) or during the NAC containing taxanes of the same TNBC patient. The pCR index was obtained by the commonly used Miller and Payne grading of breast cancer in clinic diagnosis. Grades 1–4, including no response and partial response, are considered as non-pCR. Grade 5, indicating no residual invasive cancer after treatment, is considered as pCR. Patient #26 is a non-pCR patient. Data were representative of 3 patients. (B-D) CCL20 level in sera of breast cancer patients before or during or after NAC containing taxanes was measured by ELISA. n = patient number. Scatter plots were presented according to the patients’ response to the chemotherapy. Entire cohort of patients (B), pCR patients (C), or non-pCR patients (D). Patients with unavailable pCR information were excluded in (C) and (D). *p < 0.05, by unpaired *t* test. (E) CCL20 staining by IHC in tumor sections of breast cancer patients before or after taxane-containing NAC. Scale bars: 80 μm. (F) H-Scores of CCL20 levels were presented as scatter diagram in (E). ***p < 0.001 by unpaired *t* test. (G) Cytokine antibody array was carried out with the 2-day FBS-free conditioned medium collected from MDA-MB-231 cells after treatment with TAX (13.46 nM) or DOC (14.1 nM) for 7 days. Dots labeled with red circle stand for CCL20 of duplicates. (H) Heat map was clustered with hierarchical maximum linkage and similarity metric of euclidean distance for the cytokine levels (fold change to control) in (G). (I) Similar experimental procedures as in (G) with 4T1 cells (DOC, 5 nM). (J) Heat map was clustered with the same method as in (H) for the cytokine levels from 4T1 in (I). (K) A diagrammatic sketch showing CCL20 was induced commonly in the 4 TNBC cell lines after DOC treatment and the representative patient serum (#26) during NAC. Digits in bracket indicate the number of most significantly up-regulated cytokines in the range of the defined thresholds. CCL20, C-C motif chemokine ligand 20; CTRL, control; DOC, docetaxel; ELISA, enzyme-linked immunosorbent assay; FBS, fetal bovine serum; H-Score, histo-score; IHC, immunohistochemistry; NAC, neoadjuvant chemotherapy; n.s., not significant; pCR, pathologic complete response; TAX, taxol; TNBC, triple-negative breast cancer.

Taxanes have become widely recognized as active chemotherapeutic agents in the treatment of metastatic breast cancer and early-stage breast cancer [15]. Cytokine changes during chemotherapy regulate disease progression, favoring the growth and proliferation of tumor cells [16]. However, the involvement of taxane-induced cytokines in chemoresistant breast cancer has not been well studied. Here, we investigated cytokine levels in sera from 3 TNBC patients undergoing taxane-containing NAC. Utilizing cytokine arrays, we found that NAC significantly induced a serial of cytokines in the sera of patients, including CCL20 (Fig 1A).

In order to investigate whether CCL20 is one of the commonly taxane-induced cytokines, more serum samples were collected from breast cancer patients in clinic before, during, or after chemotherapy in the presence of taxol or docetaxel in the neoadjuvant setting, and an enzyme-linked immunosorbent assay (ELISA) was conducted to measure CCL20 levels in sera. The results showed that there is no significant difference among 3 collection time points in the entire cohort of patients (Fig 1B). The patients were classified according to pCR or non-pCR. In pCR patients, there was no significant difference in the level of CCL20 at the 3 collection time points (Fig 1C). However, in non-pCR patients, CCL20 was obviously increased in the sera collected during the chemotherapy compared with that collected before NAC. CCL20 was also augmented in sera after chemotherapy compared with that collected before NAC in non-pCR patients; however, the difference was not significant, probably because of the interval time between therapy termination and blood collection (Fig 1D). These results suggest that there might be a correlation between CCL20 induction and taxane resistance. In order to verify our conjecture, we collected 36 pairs of breast tumor tissues before and after taxane-containing NAC from the same non-pCR patients in the clinical pathology from 4 cancer hospitals in China, and results showed that CCL20 staining was significantly increased in the tumor specimens from postchemotherapy, which represent the chemoresistant tumors (Fig 1E and 1F).

It is critical to investigate the effect of taxane on inducing cytokines from resistant cancer cells to clarify the relationship between chemotherapy-induced cytokines and taxane resistance. Cytokine antibody array was conducted in MDA-MB-231 cells (Fig 1G and 1H). CCL20 level was obviously elevated after taxane treatment. Similar results were also validated in human TNBC cell lines SUM149 and SUM159 and mouse TNBC cell line 4T1 (S1A, S1B, S1C and S1D Fig; Fig 1I and 1J). Then, we analyzed the induced cytokines from the 4 TNBC cell lines and the serum of patients with taxane chemotherapy. Interestingly, we found that CCL20 was the most universally and significantly up-regulated in common (Fig 1K; S1E Fig), suggesting CCL20 might play a vital role in regulating the taxane resistance of breast cancer cells. Taken together, we identified a taxane-induced cytokine CCL20, and its expression level in serum and tumor tissue showed a positive correlation with the poor pathologic response to NAC in breast cancer patients.

### Taxanes triggered CCL20 induction significantly both in vitro and in vivo

**Fig 2:**
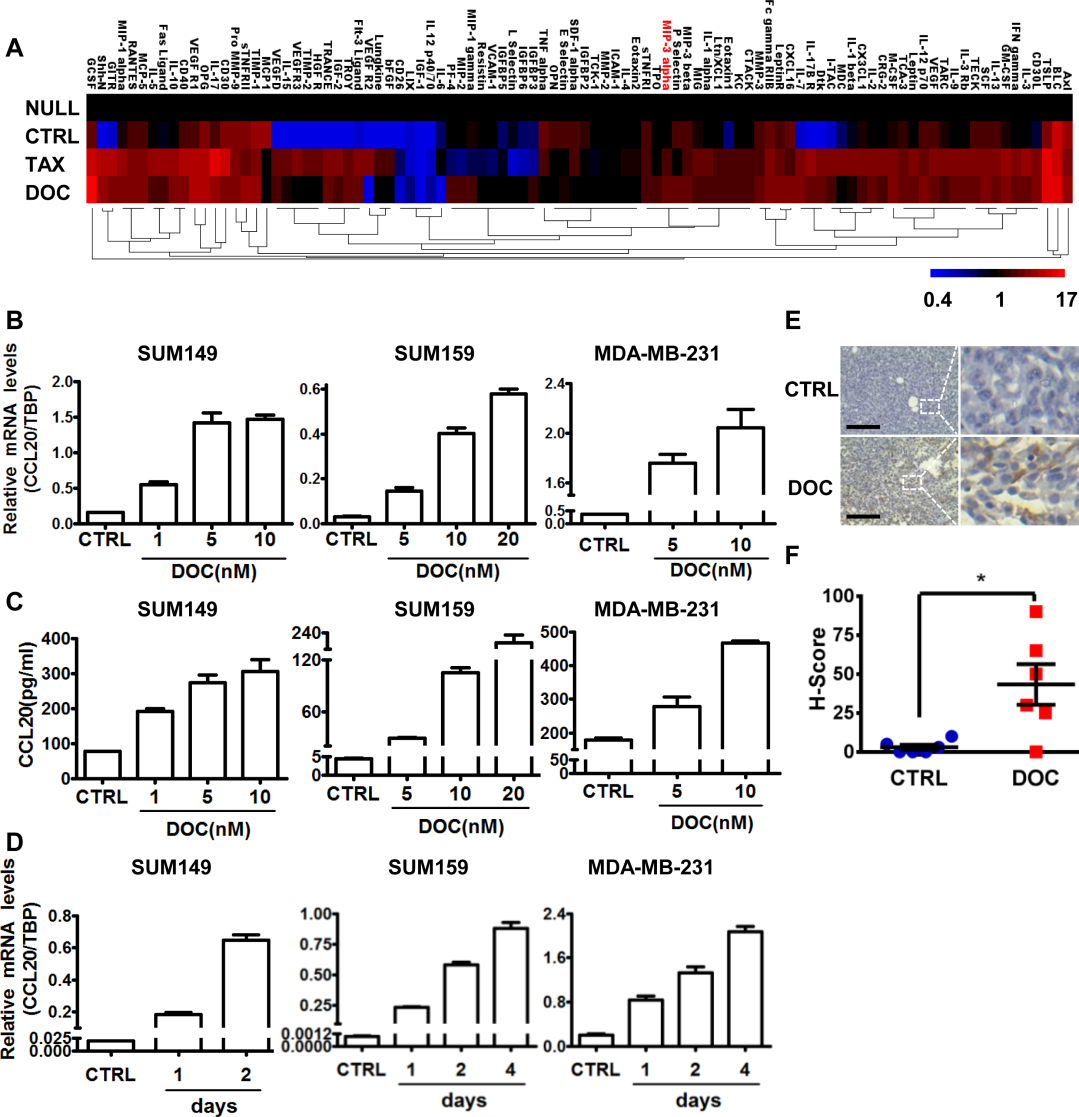
CCL20 was induced by taxanes significantly both in vitro and in vivo. (A) Sera from wild-type BALB/c (“NULL”), 4T1 tumor-bearing BALB/c mice (1 × 10^5^ 4T1 cells were injected per site of fourth mammary fat pads) without chemotherapy (CTRL) or with TAX (10 mg/kg) or DOC (10 mg/kg) i.p. once a week (the treatment was started when the average tumor size reached 2 mm in diameter). Five mice per group. After 4-week treatment, mouse cytokine antibody array was carried out with the collected sera from each group of mice, and a heat map was presented with the average cytokine levels (fold change over NULL group) of duplicates clustered with hierarchical maximum linkage and similarity metric of euclidean distance. CCL20 (MIP-3 alpha) is marked in red. (B) SUM149, SUM159, and MDA-MB-231 (MDA-231) cells were treated with the indicated concentrations of DOC for 3 days; then, qRT-PCR was performed to determine the mRNA levels of CCL20 in triplicates. (C) ELISA was carried out with 2-day FBS-free conditioned medium after treatment with the indicated concentrations of DOC for 3 days in SUM149, SUM159, and MDA-MB-231 cells. Bar graphs are representative of duplicated experiments of ELISA and 3 repeats in each experiment. (D) Breast cancer cells were treated with DOC (1 nM for SUM149, 5 nM for SUM159, 14.1 nM for MDA-MB-231) for the indicated days; then, qRT-PCR was performed to determine the mRNA levels of CCL20 in triplicates. (E) CCL20 expression was determined by IHC in the tumor sections of MDA-MB-231 xenografts (1.5 × 10^6^ MDA-MB-231 cells were injected per site of fourth mammary fat pads of nude mice) without (CTRL) or with DOC administration (10 mg/kg, i.p.) once a week for a total 5 weeks. Scale bars: 200 μm. (F) H-Scores of CCL20 levels in (E) were presented as scatter diagram. *p < 0.05 by unpaired *t* test. Tumor numbers *n* = 6 per group. Data of bar graphs were shown as mean ± SEM. CCL20, C-C motif chemokine ligand 20; CTRL, control; DOC, docetaxel; ELISA, enzyme-linked immunosorbent assay; FBS, fetal bovine serum; H-Score, histo-score; i.p., intraperitoneally; MIP-3 alpha; macrophage inflammatory protein 3 alpha; qRT-PCR, quantitative real-time PCR; TAX, taxol; TBP, TATA-box binding protein.

Next, we established xenografts with mouse breast cancer cell line 4T1 in BALB/c mice and conducted the taxane treatment in mice to mimic the clinical setting. In comparison to the tumor-free mice (“NULL”), only a few cytokines were elevated in the sera of mice bearing tumors but without treatments (“CTRL”). However, consistent with results from breast cancer patient serum, cytokine levels in the mouse sera were significantly changed when receiving the taxane treatment, among which CCL20 was obviously observed (Fig 2A). These results further suggested that the CCL20 was significantly induced in tumor-bearing mice under taxane treatment. Furthermore, we investigated whether the CCL20 induction depended on the taxane dosage or treatment duration. Utilizing 3 TNBC cell lines (SUM149, SUM159, and MDA-MB-231), we showed that CCL20 was induced by docetaxel in a dose-dependent way as measured by both quantitative real-time PCR (qRT-PCR) and ELISA (Fig 2B and 2C). Also, docetaxel treatment triggered CCL20 induction significantly, and the induction efficacy progressed in a time-dependent manner (S2A and S2B Fig; Fig 2D). In addition, we found that CCL20 was elevated in the tumor residue of MDA-MB-231 xenograft tumor model in the docetaxel treatment group, in contrast to the control group, as analyzed by immunohistochemistry (IHC) staining (Fig 2E and 2F). In brief, these results suggested that CCL20 was indeed significantly increased after taxane treatment both in vitro and in vivo.

### CCL20 promoted tumor progression in TNBC and enhanced the chemoresistance of cancer cells to taxanes

**Fig 3:**
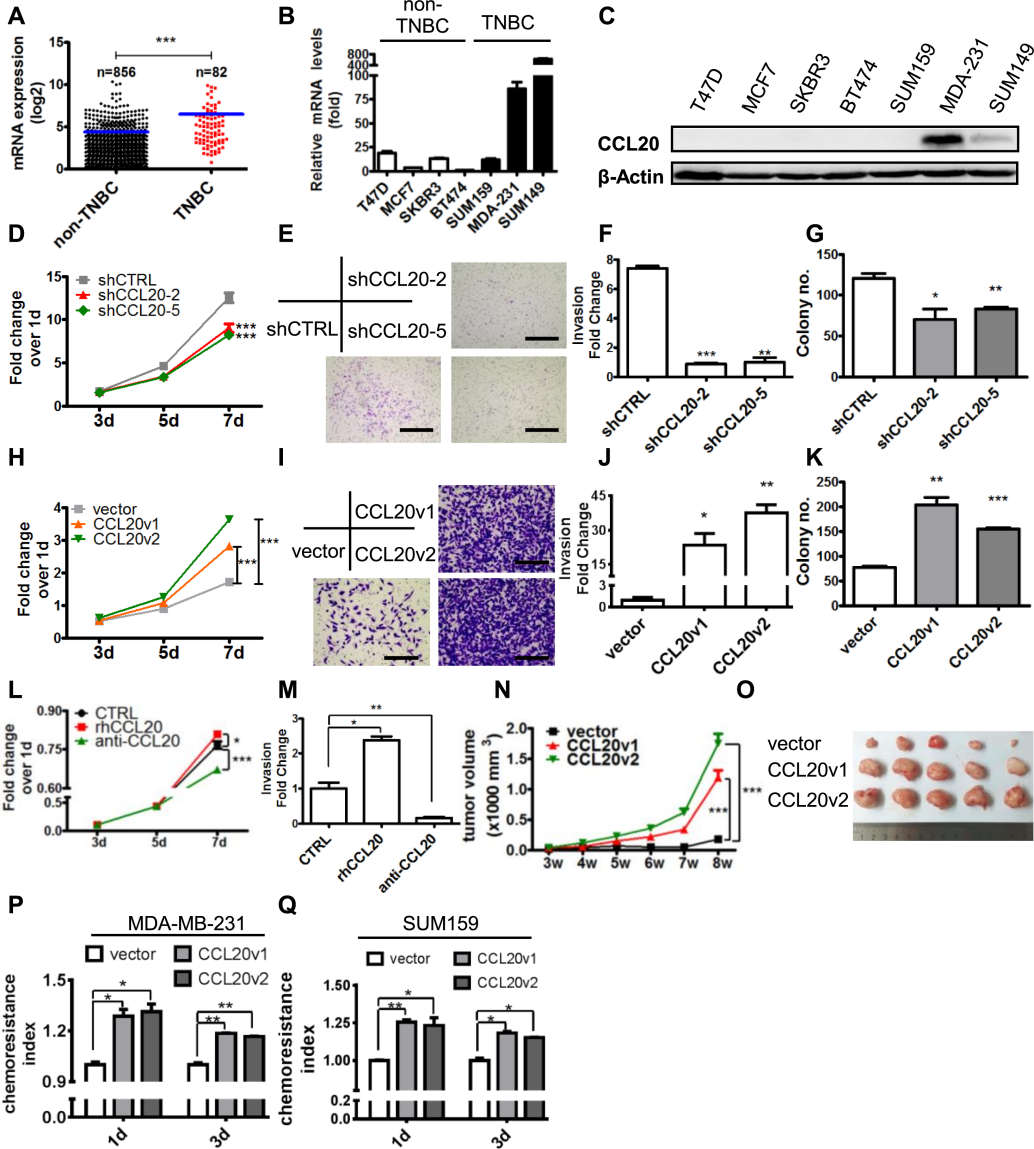
CCL20 promoted tumor progression and enhanced the chemoresistance to taxanes in TNBC. (A) CCL20 level in breast cancer tissues based on the RNA-seq data. Result here was in whole or part based upon data generated by the TCGA Research Network: http://cancergenome.nih.gov/. (B-C) CCL20 levels determined by qRT-PCR or western blot in 7 breast cancer cell lines. (D) MTT assay conducted with MDA-MB-231 cells. shCCL20-2 and shCCL20-5: two CCL20-shRNA. (E) Matrigel invasion assay conducted with MDA-MB-231. Scale bars: 400 μm. (F) Quantitative analysis of total invaded cells in (E). (G) Soft agar colony formation assay performed with MDA-MB-231 cells, and colony numbers were quantified. (H-K) MTT assay, Matrigel invasion assay, and soft agar colony formation assay were carried out with vector control or CCL20-overexpressing MDA-MB-231 cells with the same methods as described in (D-G). Scale bars: 200 μm. (L) MTT assay conducted with MDA-MB-231 cells in the presence or absence of rhCCL20 (10 ng/ml) or anti-CCL20 (200 ng/ml). (M) Matrigel invasion assay conducted in 3 groups as in (L) and quantitative analysis of total invaded cells. (N-O) Nude mice were injected with 1 × 10^6^ vector or CCL20-overexpressing MDA-MB-231 at fourth mammary fat pads. Tumor size was monitored once a week (N), and tumor image was shown after mice were killed (O). Five tumors per group. The experiments were repeated at least twice. (P-Q) Vector control or CCL20-overexpressing MDA-MB-231 (P) and SUM159 (Q) were treated with DOC (MDA-MB-231, 14.10 nM; SUM159, 10 nM) for indicated days, and the chemoresistance was analyzed as described in evaluation of chemoresistance index. Data were shown as mean ± SEM and are representative of 3 individual experiments. *p < 0.05, **p < 0.01, ***p < 0.001 by unpaired *t* test of triplicates and multiple comparisons test of 2-way ANOVA (D, H, L, and N). anti-CCL20, CCL20 neutralization antibody; CCL20, C-C motif chemokine ligand 20; DOC, docetaxel; MTT, 3-(4,5-dimethylthiazol-2-yl)-2,5 diphenyl tetrazolium bromide; qRT-PCR, quantitative real-time PCR; rhCCL20, recombinant human CCL20; RNA-seq, RNA sequencing; shCTRL, scramble control; shRNA, short hairpin RNA; TCGA, The Cancer Genome Atlas; TNBC, triple-negative breast cancer.

To determine if CCL20 regulates breast cancer malignancy, firstly, we analyzed the database of The Cancer Genome Atlas (TCGA, https://cancergenome.nih.gov/) and found that CCL20 was highly expressed in TNBC in contrast to non-TNBC (Fig 3A), which was also observed at both mRNA and protein levels in breast cancer cell lines (Fig 3B and 3C). These studies indicate CCL20 expression is positively correlated with the malignancy of breast cancer. In order to investigate the function of CCL20 in TNBC, we silenced CCL20 in TNBC cell lines and found knockdown of CCL20 significantly decreased the proliferation of cancer cells (S3A and S3B Fig; Fig 3D). Cell invasion through basement membrane protein (Matrigel) (Fig 3E and 3F) and anchorage-independent growth (Fig 3G) were also reduced in CCL20 knockdown MDA-MB-231 cells. Moreover, we established CCL20-overexpressing cell lines with 2 different isoforms of CCL20 (CCL20v1 and CCL20v2), which well simulates the physiological level of CCL20 induced by docetaxel (S3C, S3D, S3E and S3F Fig), and it was shown that CCL20 overexpression increased cell proliferation (Fig 3H; S3G Fig), the invasion ability (Fig 3I and 3J; S3H and S3I Fig), and the anchorage-independent growth (Fig 3K; S3J and S3K Fig) significantly in both MDA-MB-231 and SUM159 cells. In addition, recombinant human CCL20 (rhCCL20) and CCL20 neutralization antibody (anti-CCL20) were utilized to mimic the function-loss or function-gain assay above, and it was shown that cell proliferation and invasion were increased by rhCCL20 and decreased by anti-CCL20 in MDA-MB-231 and SUM159 cells (Fig 3L and 3M; S3L and S3M Fig), which further confirmed the role of CCL20 in regulating TNBC cell malignancy.

Our results in vivo also showed that CCL20 significantly accelerated tumor growth of MDA-MB-231 xenograft in nude mice (Fig 3N and 3O). CCL20 can be induced by taxane and promoted the development of breast cancer, which, considering the relationship between CCL20 and pCR (Fig 1D), prompted us to further observe impact of CCL20 on chemotherapy resistance. Our results showed that CCL20 overexpression promoted resistance to docetaxel in TNBC cell lines (Fig 3P and 3Q). In conclusion, CCL20 significantly promoted the progression of TNBC both in vitro and in vivo, and it was preliminarily shown that CCL20 could promote the resistance of cancer cells to taxane.

### CCL20 enhanced the taxane resistance of TNBC through promoting aldehyde dehydrogenase (ALDH^+^) population of breast cancer cells

**Fig 4:**
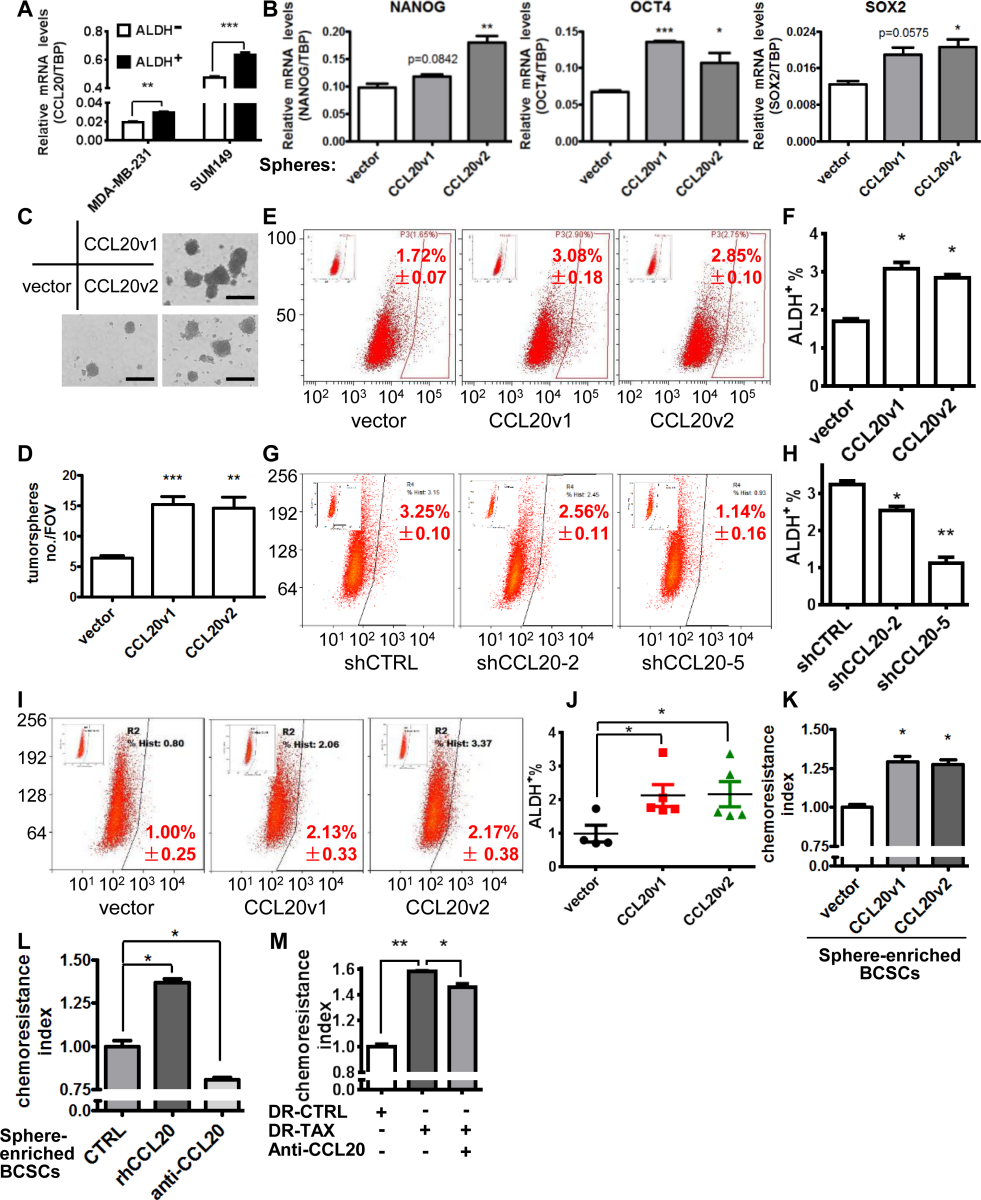
CCL20 enhanced the taxane resistance of TNBC through promoting ALDH^+^ BCSCs. (A) CCL20 levels were determined by qRT-PCR in the ALDH^+^ and ALDH^−^ cells sorted from MDA-MB-231 and SUM149 with Aldefluor Assay by flow cytometry. (B) Levels of stemness genes determined by qRT-PCR in mammospheres formed by vector or CCL20-overexpressing MDA-MB-231 cells. (C-D) Tumorsphere formation assay was conducted with vector or CCL20-overexpressing MDA-MB-231 cells (1 × 10^5^ cells/ml Mammocult Medium). Representative images were shown (C), and bar graph showed the sphere numbers per field (×40) based on randomly selected 5 fields (D). Scale bars: 400 μm. (E-H) Percentage of ALDH^+^ BCSC population in MDA-MB-231 cells after CCL20 overexpression (E, F) or knockdown (G, H) was determined by flow cytometry. (I-J) Tumors from experiments of Fig 3O were minced and digested into single cells to analyze the ALDH^+^ BCSC population by flow cytometry (I). Scatter plot showed statistics (J). Five tumors per group (except for the vector group in which 1 tumor was too small to obtain enough single cells for flow cytometry). (K) The cultured tumorspheres of MDA-MB-231 were digested to single cells and treated with DOC (14.10 nM) for 24 hours, and the chemoresistance was analyzed subsequently. (L) Single cells digested from cultured tumorspheres of MDA-MB-231 cells were treated with DOC (14.10 nM) in the absence (CTRL) or presence of rhCCL20 (10 ng/ml) or anti-CCL20 (200 ng/ml) for 24 hours, and the chemoresistance was analyzed subsequently. (M) MDA-MB-231 cells of taxol-resistant (DR-TAX) and parental (DR-CTRL) were treated with taxol (13.46 nM) in the presence or absence of anti-CCL20 (200 ng/ml) for 3 days, and then the resistance to taxol was analyzed by flow cytometry. Data were shown as mean ± SEM and are representative of 3 independent experiments. *p < 0.05, **p < 0.01, ***p < 0.001 by unpaired *t* test. ALDH, aldehyde dehydrogenase; anti-CCL20, CCL20 neutralization antibody; BCSC, breast cancer stem cells; CCL20, C-C motif chemokine ligand 20; CTRL, control; DOC, docetaxel; FOV, field of view; qRT-PCR, quantitative real-time PCR; rhCCL20, recombinant human CCL20; shCTRL, scramble control; TBP, TATA-box binding protein; TNBC, triple-negative breast cancer.

Others and we have shown that BCSCs are enriched after chemotherapy treatment [17] (S4A Fig), which indicates taxane-resistant BCSCs limit the efficacy of taxane therapy. To explore the regulation of BCSCs via CCL20, we first investigated the differential expression of CCL20 in BCSCs defined by ALDH activity and found there was higher level of CCL20 in flow-sorted ALDH^+^ population than in ALDH^−^ population in TNBC (Fig 4A). Moreover, the receptor of CCL20, C-C motif chemokine receptor type 6 (CCR6), was also up-regulated in ALDH^+^ BCSCs of SUM149, MDA-MB-231, and PDX (S4B and S4C Fig). These indicate the probable role of CCL20 in promoting BCSC stemness property. Hence, the stemness and self-renewal of BCSCs were determined after CCL20 overexpression. Stem cell genes NANOG, OCT4, and SOX2 were up-regulated in CCL20-overexpressing cells (Fig 4B; S4D Fig), which suggested that CCL20 promoted the stem property of BCSCs. Moreover, the tumorsphere formation was evidently increased in CCL20-overexpressing cells in comparison to the control cells (Fig 4C and 4D; S4E and S4F Fig), suggesting the increase of self-renewal of BCSCs. Furthermore, enhancement of BCSC properties mediated via CCL20 affected the ALDH^+^ population, as shown in that CCL20 overexpression evidently increased ALDH^+^ cells, leading to the population expansion of BCSCs (Fig 4E and 4F), which was reversed by CCL20 silencing (Fig 4G and 4H). The promotion of CCL20 on expansion of ALDH^+^ cells was also proved in the MDA-MB-231 xenograft model in vivo, and the results showed that the ALDH^+^ population in CCL20-overexpressing xenografts was increased by 1-fold (Fig 4I and 4J).

Next, we tested if the effect of CCL20 on regulating BCSC expansion leads to taxane resistance. Utilizing in vitro sphere-culture system to enrich BCSCs, we showed that both CCL20 overexpression and rhCCL20 treatment increased the docetaxel resistance of sphere-formed cells, while the anti-CCL20 blocked this effect (Fig 4K and 4L). It is interesting that, after blocking CCL20, the drug resistance of the taxol-resistant subline of MDA-MB-231 was reduced to some extent (Fig 4M). Taken together, CCL20 drives the self-renewal and expansion of ALDH^+^ population to promote the properties of BCSCs, leading to the increased taxane resistance of breast cancer cells.

### CCL20 enhances the taxane resistance of TNBC cells through PKCζ- and p38 MAPK-mediated NF-κB activation

**Fig 5:**
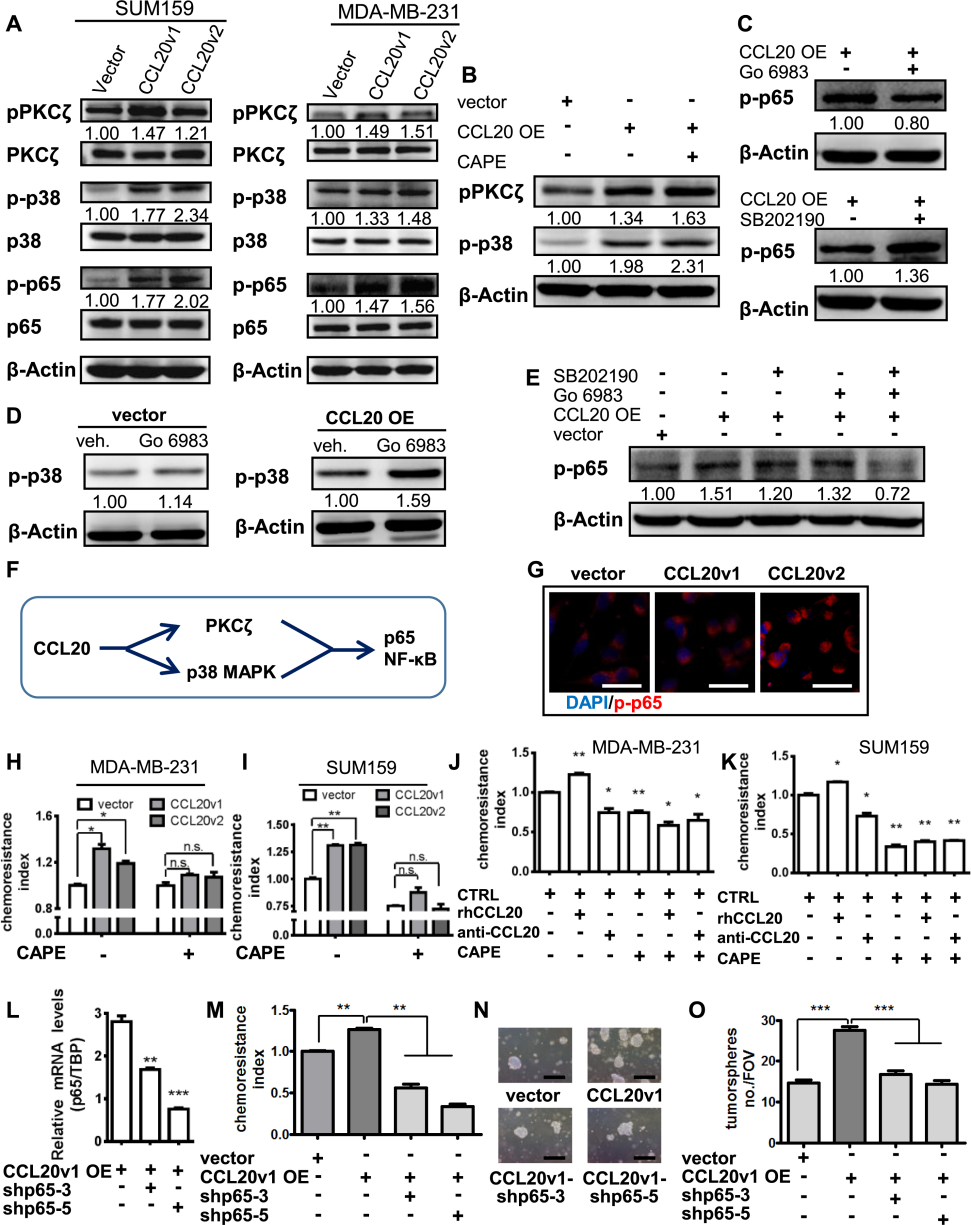
CCL20 enhances the taxane resistance of TNBC cells through PKCζ- and p38 MAPK–mediated NF-κB activation. (A) After FBS starvation for 24 hours, western blot was conducted with SUM159 and MDA-MB-231 cells. The immunoblotting bands were quantified, normalized with β-actin, and fold-changed to the first panel (similarly hereinafter). (B) Vector and CCL20v1 OE MDA-MB-231 cells were cultured in the presence or absence of specific inhibitor of p65 NF-κB activation (CAPE, 5 μM) under FBS starvation conditions for 12 hours, and western blot was performed. (C) FBS-starved CCL20v1 OE MDA-MB-231 cells were treated with PKCζ inhibitor (Go 6983, 5 μM) or p38 MAPK inhibitor (SB202190, 20 μM) for 12 hours and immunoblotted. (D) Go 6983 (5 μM) or vehicle (DMSO) was used to treat vector and CCL20v1 OE MDA-MB-231 for 12 hours under FBS starvation, and immunoblotting was performed. (E) FBS-starved vector and CCL20v1 OE SUM159 were treated (Go 6983, 5 μM; SB202190, 20 μM) for 12 hours and immunoblotted. (F) Illustration of the CCL20-PKCζ/p38-NF-κB axis. (G) Images of immunofluorescence performed with MDA-MB-231 were captured with confocal microscope. Blue, DAPI for nucleus; red, phosphorylated p65 NF-κB. Scale bars: 40 μm. (H-I) Single cells dissociated from mammospheres of MDA-MB-231 (H) or SUM159 (I) were treated with DOC (MDA-MB-231, 14.10 nM; SUM159, 5 nM) in the absence or presence of CAPE (5 μM) for 24 hours, and the chemoresistance was analyzed. (J-K) Single cells dissociated from mammospheres of MDA-MB-231 (J) or SUM159 (K) were treated with DOC (MDA-MB-231: 14.10 nM; SUM159: 5 nM) in the absence or presence of rhCCL20 (10 ng/ml), anti-CCL20 (200 ng/ml), or CAPE (5 μM) for 24 hours, and the chemoresistance was analyzed. The subsequent bars were compared to the first bar (control) to conduct statistics. (L) p65 knockdown verified by qRT-PCR in CCL20v1 OE MDA-MB-231 cells. (M) Single cells dissociated from MDA-MB-231 tumorspheres were treated with DOC (14.10 nM) for 24 hours and subjected to chemoresistance analysis. (N-O) Tumorsphere formation assay performed with MDA-MB-231 (N) and statistics (O). Scale bars: 400 μm. Data are representative of at least 3 independent experiments and shown as mean ± SEM. *p < 0.05, **p < 0.01, ***p < 0.001 by unpaired *t* test of triplicates. anti-CCL20, CCL20 neutralization antibody; CAPE, caffeic acid phenethyl ester; CCL20, C-C motif chemokine ligand 20; DOC, docetaxel; FBS, fetal bovine serum; FOV, field of view; Go, Gene Ontology; MAPK, mitogen-activated protein kinase; NF-κB, nuclear factor kappa B; OE, overexpressing; PKCζ, protein kinase Cζ; qRT-PCR, quantitative real-time PCR; rhCCL20, recombinant human CCL20; TBP, TATA-box binding protein; TNBC, triple-negative breast cancer.

CCL20 is the only ligand that activates CCR6 [18]. In thyroid cancer, CCL20/CCR6 promotes the invasion and migration of thyroid tumor cells via p65 NF-κB signaling [19]. The intermediate mechanism of NF-κB activation via CCL20 still remains unknown. As the sole receptor for CCL20 [18], CCR6 belongs to the superfamily of G protein–coupled receptor (GPCR), which provides signal-coupling mechanisms to heptahelical cell surface receptors and is critically involved in the regulation of the MAPK pathway [20]. Moreover, GPCR-coupled G protein can act as an adaptor protein to activate PKCζ in NIH 3T3-m1R cells [21]. Here in our study, after overexpression of CCL20, an increase in p65 NF-κB activation by phosphorylation was observed in breast cancer cells. The activation of p38 MAPK and PKCζ via phosphorylation was also increased by CCL20 overexpression in breast cancer cells (Fig 5A). These results prompted us to demonstrate the probable mechanisms of CCL20 signal transduction in regulating the chemoresistance of BCSCs.

As the downstream role of p65 NF-κB in transducing signal, whether p65 activation was mediated by activity of p38 MAPK and PKCζ was explored. We found that the pharmacological inhibition of p65 NF-κB activation via the specific inhibitor caffeic acid phenethyl ester (CAPE) did not affect the increased activity of p38 MAPK or PKCζ caused by CCL20 overexpression (Fig 5B, S5A Fig), which suggested that p65 probably was the downstream effector of p38 MAPK or PKCζ when transducing CCL20 stimuli. But phosphorylation of p65 was not substantially decreased by inhibition of either PKCζ alone (Gene Ontology [Go] 6983) or p38 MAPK alone (SB202190) (Fig 5C, S5B Fig). In addition, it was noticed that PKCζ inhibition actually increased the activation of p38 MAPK in CCL20-overexpressing cells, which was not observed in vector cells (Fig 5D), partly accounting for the reason why effect of PKCζ inhibition alone on p65 activation was limited. Interestingly, the inhibition of both PKCζ and p38 MAPK significantly abolished the enhancement of p65 NF-κB activity induced by CCL20 (Fig 5E, S5C Fig). These results suggested that p38 or PKCζ mediated p65 NF-κB activation in parallel manner under CCL20 overexpression (Fig 5F).

CCL20 activated p38 MAPK or PKCζ, followed by a dramatic increase of the phosphorylated p65 NF-κB translocating into the nucleus, which indicates the activation of NF-κB pathway (Fig 5G). Whether p65 NF-κB activation mediates the CCL20-induced chemoresistance was determined, and we found that the enhancement of CCL20 on docetaxel resistance was abolished by CAPE in the BCSC-enriched mammosphere-derived cells from both MDA-MB-231 (Fig 5H) and SUM159 (Fig 5I). The docetaxel resistance enhanced by rhCCL20 in the BCSC-enriched mammosphere-derived cells from both MDA-MB-231 (Fig 5J) and SUM159 (Fig 5K) was also abolished by CAPE. In addition, the degree of PKCζ or p38 blockage alone was limited for the remission of resistance, and the combinatorial inhibition of the two significantly reduced chemoresistance induced by CCL20 (S5D and S5E Fig). This further indicates that PKCζ and p38 mediate CCL20-induced drug resistance in a parallel manner, consistent with their impacts on the activation of p65 NF-κB (Fig 5E).

To further confirm these findings, p65 was knocked down in CCL20v1-overexpressing breast cancer cells (Fig 5L), and we found that docetaxel resistance of CCL20v1-overexpressing cancer cells was also abolished by p65 knockdown (Fig 5M). Furthermore, we also noticed that the tumorsphere-forming ability of CCL20v1-overexpressing breast cancer cells was inhibited by p65 silencing (Fig 5N and 5O). In addition, the p65 silence alone significantly reduced the chemoresistance and tumorsphere formation of BCSCs, further confirming the role of NF-κB in this process (S5F, S5G, S5H, S5I, S5J and S5K Fig). And the CCL20-dependent increase in the percentage of ALDH^+^ cells is mediated through p65 NF-κB (S5L Fig).

These studies suggested that the activation of p65 NF-κB pathway induced by CCL20 was mediated by p38 MAPK and PKCζ activity, respectively, and p65 NF-κB activation plays an important role in mediating CCL20-promoted taxane resistance of BCSCs.

### ABCB1 up-regulation mediated by CCL20-induced NF-κB activation reduced the intracellular abundance of taxane and promoted chemoresistance in breast cancer cells

**Fig 6:**
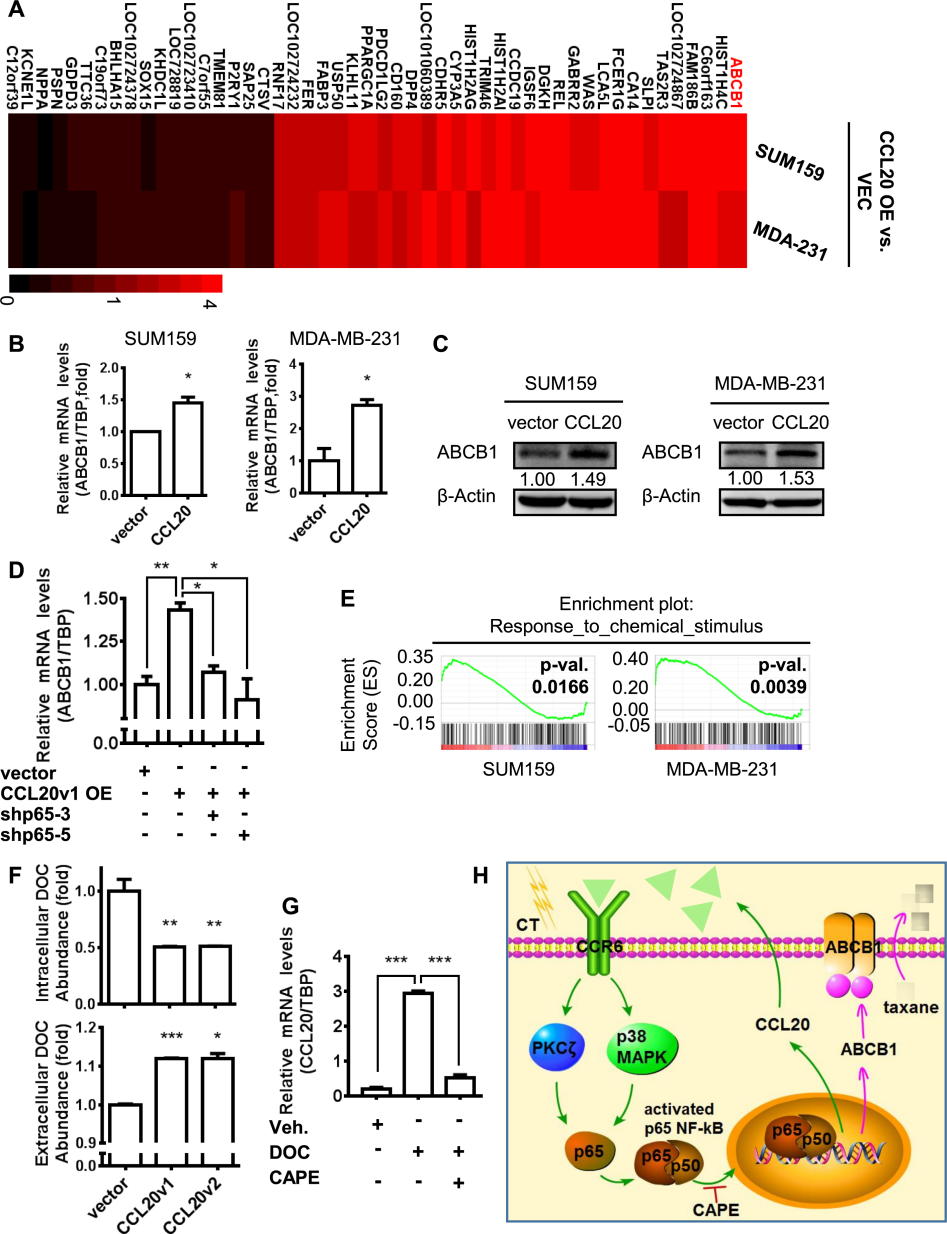
ABCB1 up-regulation mediated by CCL20-induced NF-κB activation reduced the intracellular abundance of taxane and promoted chemoresistance in breast cancer cells. (A) Transcriptome sequencing (RNA-seq) utilizing the next-generation technology was conducted with RNA from vector (“VEC”) and CCL20v1-overexpressing (“CCL20 OE”) SUM159 cells and MDA-MB-231 cells. Heat map shows expression levels of the most up-regulated genes (>1.5-fold) and significantly down-regulated genes (decline more than 40%) both in CCL20v1-overexpressing cells of SUM159 and MDA-MB-231 versus vector. Values are the fold change to vector. (B-C) The mRNA and protein levels of ABCB1 were determined by qRT-PCR or western blot in vector and CCL20v1-overexpressed cells of SUM159 and MDA-MB-231. (D) Level of ABCB1 was determined by qRT-PCR in MDA-MB-231 cells. (E) Pathway of response to chemical stimulus was positively correlated with CCL20 overexpression in GSEA from RNA-seq in (A). Nominal *P* value was shown. (F) Vector and CCL20-overexpressing MDA-MB-231 cells were treated with 200 nM DOC for 12 hours. Then, supernatants and cells were collected for quantitative analysis of DOC abundance with HPLC-MS. (G) MDA-MB-231 cells were cultured in the presence or absence of DOC (10 nM) or CAPE (5 μM); then, the mRNA level of CCL20 was determined by qRT-PCR. (H) A cartoon to illustrate the pattern of CCL20-induced NF-κB activation through PKCζ or p38 MAPK pathway to up-regulate expression of ABCB1 for taxane pumping-out, leading to chemoresistance. Data are shown as mean ± SEM and are representative of 3 independent experiments. *p < 0.05, **p < 0.01, ***p < 0.001 by unpaired *t* test of triplicates. ABCB1, ATP-binding cassette subfamily B member 1; CAPE, caffeic acid phenethyl ester; CCL20, C-C motif chemokine ligand 20; CCR6, C-C motif chemokine receptor type 6; DOC, docetaxel; GSEEA, Gene Set Enrichment Analysis; HPLC-MS, high-performance liquid chromatography–mass spectrometry; MAPK, mitogen-activated protein kinase; NF-κB, nuclear factor kappa B; PKCζ, protein kinase Cζ; qRT-PCR, quantitative real-time PCR; RNA-seq, RNA sequencing; TBP, TATA-box binding protein.

Differential and diverse transcription control via NF-κB appears to be context-dependent and stimulus-specific regulation of gene expression [22,23]. Alteration of transcriptional events after CCL20-induced p65 NF-κB activation has not been studied yet. RNA sequencing (RNA-seq) was used to observe the transcriptome change by overexpression of CCL20 in TNBCs. Our results showed that the expression levels of the 32 genes were up-regulated by 1.5-fold or more, and 18 genes were down-regulated by 40% or more with CCL20 overexpression both in SUM159 and MDA-MB-231 cells (Fig 6A), including ABCB1. Up-regulation of ABCB1 transcription after CCL20 overexpression was validated by qRT-PCR (Fig 6B) and western blot (Fig 6C). Similar to CCL20 expression pattern (Fig 4A), ABCB1 was highly expressed in ALDH^+^ cells, compared with ALDH^−^ cells (S6A and S6B Fig). In addition, the CCL20-induced up-regulation of ABCB1 can be abrogated by p65 silencing, suggesting that p65 NF-κB mediated the increased transcription of ABCB1 (Fig 6D).

ABCB1, a member of the superfamily of ABC transporters, is an active efflux pump for a variety of toxins as well as dietary and environmental carcinogens and drugs [24]. More interestingly, the Gene Set Enrichment Analysis (GSEA) showed that the pathway termed response to chemical stimulus, which ABCB1 was in, was positively correlated with CCL20 overexpression significantly, indicating CCL20 enriches the gene sets with resistant ability for cancer cells to respond to chemical drugs (Fig 6E). Therefore, we observed the pharmacological distribution and abundance of docetaxel in breast cancer cells after overexpression of CCL20 by high-performance liquid chromatography–mass spectrometry (HPLC-MS) (S6C Fig). Our results showed that, in comparison to vector cells, the intracellular drug abundance in CCL20-overexpressing cancer cells was decreased by 50%, and more docetaxel was accumulated in the extracellular supernatant (Fig 6F). In view of the ability of ABCB1 to transport taxane as efflux pump [25,26], the up-regulation of ABCB1 induced by CCL20 in our current studies might account for the pharmacological intracellular abundance of taxane being lower in CCL20-overexpressing cells than vector cells, which promoted the taxane resistance of breast cancer cells. In addition, the silence of ABCB1 also reversed the CCL20-induced resistance to chemotherapy (S6D Fig).

Together, these studies demonstrated that taxane-induced CCL20 activated NF-κB through PKCζ or p38 and then promoted the expression of ABCB1, leading to the chemoresistance of breast cancer cells. In addition, we were excited to observe that the increase of CCL20 induced by taxane depended on p65 NF-κB activity (Fig 6G), suggesting that the activated p65 NF-κB could regulate CCL20 level in positive feedback to further enhance the effect of CCL20 on chemoresistance. Reduction in PKCζ and p38 activation after NF-κB inhibition also further confirmed the existence of the CCL20-triggered feedback loop under docetaxel-treatment circumstances (S6E and S6F Fig). The whole regulation loop for CCL20 and BCSC chemoresistance can be demonstrated as in Fig 6H. Our current studies are the first, to our knowledge, to demonstrate the mutual regulation between CCL20 and NF-κB that up-regulates efflux pump protein ABCB1 to promote chemoresistance in BCSCs.

### CCL20 predicted poor prognosis, and the blockade to CCL20-NF-κB pathway reversed the chemoresistance of breast cancer via attenuating BCSCs

**Fig 7:**
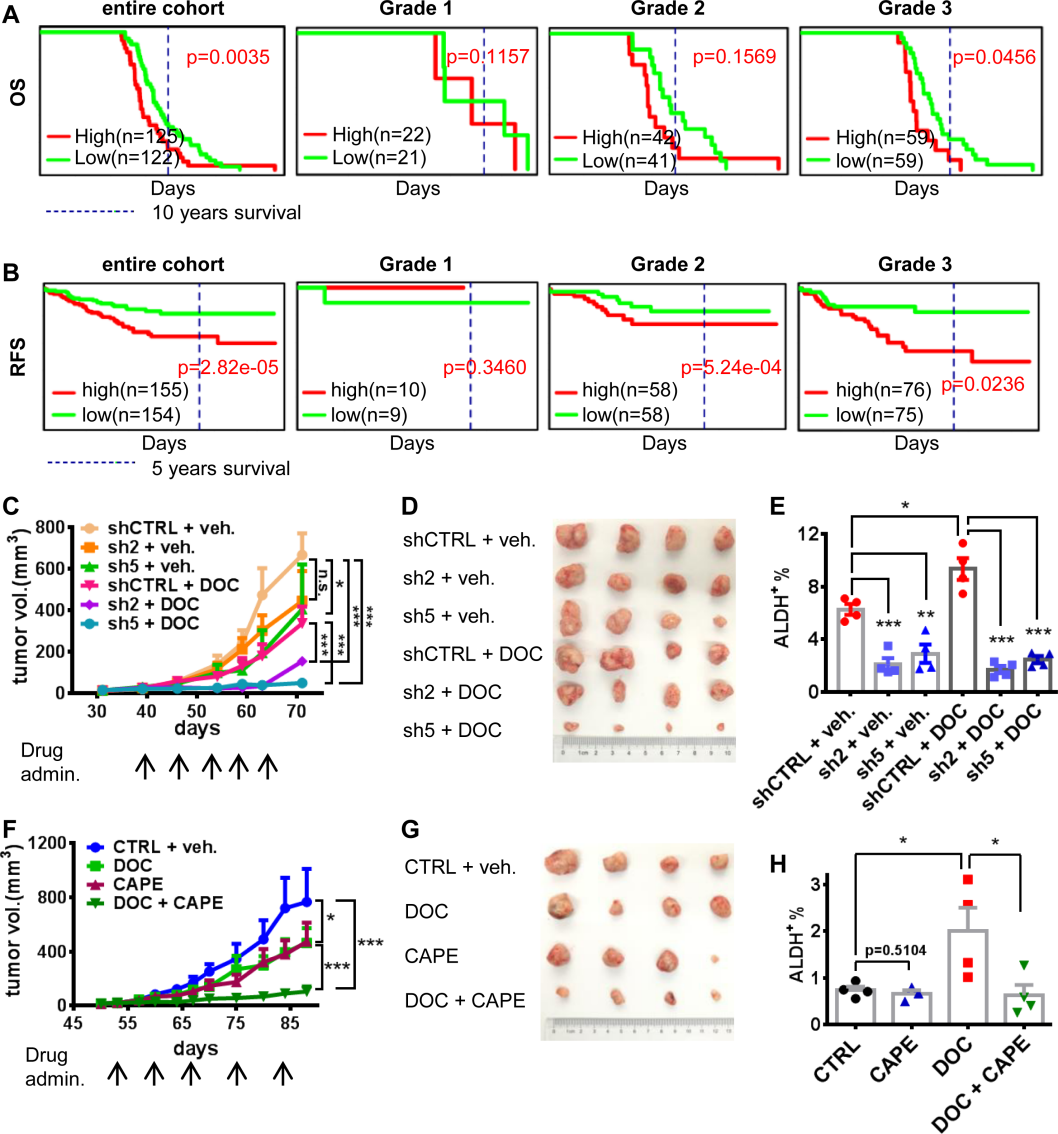
High CCL20 predicted poor prognosis of breast cancer patients, and the blockade to CCL20-NF-κB pathway reversed the chemoresistance of breast cancer. (A-B) The OS (A) and RFS (B) influenced by CCL20 expression in breast cancer from the online database (PROGgeneV2). Grades 1–3, the histological grading of breast cancer. (C-D) Nude mice were injected with 1 × 10^6^ shCTRL or CCL20-knockdown (sh2, sh5) MDA-MB-231 cells at fourth mammary fat pads. When the average tumor volume reached 20 mm^3^, mice of each group were divided into 2 new groups randomly at similar tumor volume and treated with DOC (10 mg/kg, i.p., once a week) or vehicle (“veh.”). Tumor size was monitored once a week, (C) and tumor image was taken after mice were killed (D). Tumor number *n* = 4. (E) Tumors from (D) were minced and digested into single cells for determining the ALDH^+^ BCSC population by flow cytometry. (F-G) Nude mice were injected with 1 × 10^5^ cells digested from PDX of TNBC (#USTC11 established by our group) at fourth mammary fat pads. After tumor initiation, mice were divided into 4 groups as criteria in (C) and treated with vehicle, DOC (10 mg/kg), CAPE (10 mg/kg), or both once a week i.p. Tumor size was monitored every 3 days (F), and tumor image was taken after mice were killed (G). Tumor number *n* = 4. (H) ALDH^+^ population in tumors from (G) was determined as method in (E). Four tumors per group (except CAPE group, in which 1 tumor was too small to obtain enough single cells for determination). These experiments were repeated at least twice. Data are shown as mean ± SEM. *p < 0.05, **p < 0.01, ***p < 0.001 analyzed by unpaired *t* test (E and H) or multiple comparison test of 2-way ANOVA (C and F) and log rank test (A and B). ALDH, aldehyde dehydrogenase; BCSC, breast cancer stem cell; CAPE, caffeic acid phenethyl ester; CCL20, C-C motif chemokine ligand 20; DOC, docetaxel; i.p., intraperitoneally; OS, overall survival; PDX, patient-derived xenograft; RFS, relapse-free survival; shCTRL, scramble control; TNBC, triple-negative breast cancer.

Through analyzing the cohort of basal breast cancer cases, we found that high CCL20 expression significantly predicted poor OS in the entire cohort of breast cancer patients (not only for patients treated by taxane) (Fig 7A), which, given the key role of CCL20 in modulating chemotherapeutic resistance, may provide an important clue for the design of subsequent new chemotherapy regimens for patients who have received chemotherapy but failed the treatment or are about to receive chemotherapy. Moreover, we found that the higher the histological grade, the more obvious the effect of CCL20 on the deterioration of OS. More interestingly, in the cohort of human epidermal receptor 2 (HER2)-negative breast cancer cases including TNBC or non-TNBC, after neoadjuvant taxane-containing chemotherapy, the patients with high CCL20 level were in a poor condition of relapse-free survival (RFS), and the higher the grade, the poorer the 5-year RFS in breast cancer patients with high CCL20 expression after NAC containing taxane (Fig 7B), which revealed that CCL20 played a vital role in influencing patient survival after taxane treatment not only in TNBC but also probably in non-TNBC. Our results have shown that high CCL20 level contributed to taxane resistance. The residual taxane-resistant BCSC-enriched cells after chemotherapy tended to reproliferate and lead to tumor relapse, decreasing patient probability in RFS.

CCL20 intervention may improve taxane therapy for breast cancer, including TNBCs. Our results in vivo suggested that CCL20 silencing could significantly improve the therapeutic effect of docetaxel and inhibit tumor growth in MDA-MB-231 tumor model (Fig 7C and 7D). Moreover, the enrichment of ALDH^+^ BCSCs and CD24^−^CD44^+^ BCSCs induced by docetaxel treatment in tumors can be reversed by CCL20 knockdown (Fig 7E and S7A Fig). Since we have shown that p65 NF-κB mediated the effects of CCL20, therefore, we also investigated the effect of blocking p65 NF-κB activation on the tumor growth. In the PDX model (TNBC), the inhibition of p65 NF-κB could enhance the antitumor effect of docetaxel significantly (Fig 7F and 7G). Moreover, the phenomenon of BCSC enrichment induced by docetaxel treatment was also inhibited by CAPE (Fig 7H and S7B Fig). In conclusion, high expression of CCL20 predicts worse prognosis in breast cancer patients. The combinational therapies of taxane and the blockade of CCL20 or its downstream signaling molecules can improve therapeutic effect of TNBC through attenuating BCSCs.

## Discussion

TNBC has become a worldwide threat because of its high invasiveness, recurrence, drug resistance, and lack of effective treatment prescription [27]. Taxanes are the commonly used chemotherapeutic agents for TNBC [28]. The chemotherapeutic resistance during taxane treatment seriously affected the OS of cancer patients and brought huge complications to the clinician. Thus, it is urgent to demonstrate the mechanisms such as defining the key molecules in mediating taxane resistance in treating TNBCs.

In response to the influence of repression and stimuli from chemotherapeutic agents, cancer cells will undergo multiple changes, such as alteration of the cytokine secretion. Through cytokine antibody array, we observed the correlation between CCL20 in patient serum and the indication of therapeutic response. In our studies, CCL20 emerged as the most responsive cytokine to taxane treatment, which is dosage dependent and time dependent. However, as far as the taxane stimulation on CCL20 is concerned, not only in TNBC but the phenomenon of CCL20 induction via taxane treatment also occurred in non-TNBC, actually, and the expression level of CCL20 in taxol-resistant sublines of colon cancer and ovarian cancer was significantly elevated (S8 Fig). These suggest that CCL20 may confer taxane resistance in various molecule types of breast cancer and even across multiple cancers, in which further exploration and study are needed.

Previous studies showed that CCL20 promoted proliferation and migration of normal breast epithelial cells [29,30]. Little work has revealed the regulation of CCL20 on breast cancer. Our studies showed that CCL20 stimulated the proliferation, invasion, and anchorage-independent growth in TNBC and promoted tumor outgrowth in vivo. In addition, we discovered CCL20 also exhibited strengthening in resistance to taxane, demonstrating its pivotal role in the regulation of breast cancer malignancy. And our results demonstrated for the first time that CCL20 promoted chemoresistance in BCSCs by enhancing self-renewal and stemness characteristics of BCSCs.

The range of tumor types displaying aberrant NF-κB activity is extensive and comprises many solid tumors, as well as lymphomas and leukemias [31,32]. NF-κB signaling exhibits the intricate transcriptional control of gene expression and diverse posttranslational modifications in a stimulus-specific or context-dependent way, for which the influence on the tumor progression is also complex tumor promotion or suppression, chemoresistant or chemosensitive effect, and even playing the biphasic role with dynamic regulation in tumor progression [22,33]. In our results, p65 NF-κB mediated CCL20-induced TNBC resistance to taxane. In this process, PKCζ and p38 delivered the CCL20 stimulus in parallel. We demonstrated that, under the stimulation of taxane-induced CCL20, the p65 NF-κB was mainly shown to promote resistance to apoptosis induced by taxane treatment. Combined with the inhibition of p65 NF-κB activity, the therapeutic effect of docetaxel was significantly improved in vivo, which was consistent with the elevated therapeutic efficacy under the combinational treatment of docetaxel and direct CCL20 blockage in vivo. These discoveries shed light on direct targeting CCL20 or small molecule inhibition of CCL20-induced signaling being able to potentiate chemotherapeutic efficacy in breast cancer.

In our study, one of the consequences following CCL20-induced p65 NF-κB activation was the up-regulation of ABCB1, which was involved in the efflux of a variety of drugs, including taxane [25,26]. The observation that the intracellular abundance of docetaxel was significantly decreased after overexpression of CCL20 in our study well demonstrated why CCL20-induced NF-κB activation promoted taxane resistance. Our studies offer a novel strategy to solve taxane resistance in breast cancer, including TNBC—that is, the combination of taxane treatment and the adjuvant pharmacological inhibition of ABCB1 may greatly improve the chemotherapeutic efficacy in breast cancer.

In conclusion, our results demonstrated for the first time that CCL20 promoted chemoresistance in BCSCs through the positive feedback loop mediated by PKCζ/p38-NF-κB. Importantly, our findings suggest that CCL20 can be used as a novel chemoresistance marker for the adjuvant evaluation of therapeutic efficacy in taxane treatment and as a therapeutic target to improve the efficacy of taxane therapy for TNBC patients.

## Materials and methods

### Ethics statement

Written informed consent was obtained from all patients before surgery, as advocated by the regional ethics committee. The study was approved by Fudan University Shanghai Cancer Center Institutional Review Board (050432-4-1212B), Jiangsu University Affiliated People's Hospital Ethics Committee (20150180), Research Ethical Committee of Qilu Hospital of Shandong University (BR-20111201), and the Institutional Ethics Committee of Southwest Hospital, Third Military Medical University (2015ky21).

All mouse experiments were conducted in accordance with standard operating procedures approved by the University Committee on the Use and Care of Animals at University of Science and Technology of China (USTCACUC1401020).

### Human breast cancer specimens and patient serum

All paraffin sections of tumors from breast cancer patients used in this study were provided by Fudan University Shanghai Cancer Center, the First People's Hospital of Zhenjiang City, Southwest Hospital, and Qilu Hospital. And sera from patients were obtained from Fudan University Shanghai Cancer Center.

### Cell culture and tumorigenicity in mice

Breast cancer cell line SUM149 and SUM159 were from Asterland [34]. MDA-MB-231 and 4T1 were purchased from ATCC. The culture medium for SUM149 and SUM159 is Ham’s F-12 (Invitrogen) supplemented with 5% FBS (Gibco, United States of America), 5 μg/mL insulin, and 1 ug/mL hydrocortisone (both from Sigma, St. Louis, USA) and 1% pen-strep (Beyotime, China). MDA-MB-231 and 4T1 cells were maintained in RPMI1640 medium (Gibco, USA) supplemented with 10% FBS (Gibco, USA) and 1% pen-strep antibiotic (Beyotime, China). All of the cell lines were tested and authenticated. These cell lines were maintained at 37°C in an atmosphere of 5% CO_2_.

All mice were bred and housed in AAALAC-accredited specific pathogen-free rodent facilities at University of Science and Technology of China. Mice were housed in sterilized, ventilated microisolator cages and supplied with autoclaved commercial chow and sterile water.

Tumorigenicity was determined by injecting MDA-MB-231 cells and 4T1 cells with Matrigel into fourth mammary fat pads of 1-month-old female nude mice or BALB/c mice. The animals were euthanized when the tumors were 1.0–1.5 cm in diameter. Before being euthanized, a small quantity of blood was collected into a tube without additives from mouse eye socket. Then, it was kept at RT for 20 minutes, centrifuged 10 minutes at 3,000 rpm, aliquoted into small tubes, and stored at −80°C until use. A portion of each tumor was fixed in formalin and embedded in paraffin for histological analysis. Another portion was digested to single cell and utilized for flow cytometry. The tumor sizes were measured once a week with a caliper and calculated as tumor volume = Length × Width^2^ / 2.

### Breast tumor cell isolation, flow cytometry, and sorting

Mouse tumors were dissected and minced into small pieces and resuspended with collagenase-hyaluronidase digestion reagent (Catalog #07912, STEMCELL Technologies, USA). Tumor pieces were digested for approximately 1 hour at 37°C and shaken once every 15 minutes. Cell aggregates were removed by filtering cell suspension with 40 μm filter, and ammonium chloride solution (Catalog #07850, STEMCELL Technologies, USA) was used for lysis of erythrocytes for 10 minutes at RT. Then, cell suspension was centrifuged at 1,200 rpm for 5 minutes and resuspended for subsequent experiments.

When analyzing tumor cells from xenograft of human MDA-MB-231 model or PDX, mouse cells were excluded by gating in flow cytometry using H-2K^d^-PE (Clone SF1-1.1, Cat 116607, BioLegend, USA).

Single cells from tumor or cell line were assessed for their ALDH activity using the ALDEFLUOR Kit (Catalog #01700, STEMCELL Technologies, USA) following the manufacturer’s procedures. Briefly, 1 × 10^6^ cells were resuspended in 1 ml ALDEFLUOR buffer, and activated ALDEFLUOR substrate (BAAA) was added. Immediately, 300 μl of them were separated in another tube with the inhibitor DEAB (Catalog #01705, STEMCELL Technologies, USA). The incubation was performed for 40 minutes at 37°C, and then cells were resuspended with DAPI solution for the following flow cytometry analysis. In addition, single cells from tumor or cell line were also stained with CD24 antibody (Cat 311118, BioLegend, USA) and CD44 antibody (Cat 560532, BD Pharmingen, USA) for 30 minutes on ice and then resuspended with DAPI solution for flow cytometry analysis. A population of 10,000 living cells was captured and analyzed, and all fluorescence-activated cell sorting (FACS) experiments was performed using MOFLO ASTRIOS flow cytometer system (BECKMAN COULTER, USA) and software Summit 6.1. Gating was based on "Fluorescence Minus One" controls.

### Evaluation of chemoresistance index

After cells were treated with taxol or docetaxel, they were collected to determine apoptosis with staining APC-Annexin V (550474, BD Pharmingen) following the manufacturer’s recommendations. When carrying on flow cytometry, a population of 10,000 for all events was captured, and apoptosis population was analyzed by MOFLO ASTRIOS flow cytometer system (BECKMAN COULTER, USA) and software Summit 6.1. High apoptosis indicates low ability of chemoresistance to taxane, and apoptosis defined by APC-positive population was switched to Chemoresistance Index according to the formula 1-(Test-Ctrl)/Ctrl. Ctrl is the contrast group that other groups were fold-changed to.

### Mammosphere formation assay and sphere digestion

Human breast tumor cells were cultured with MammoCult Human Medium Kit (#05620, STEMCELL Technologies, USA) supplemented with 4 μg/mL Heparin (#07980, STEMCELL Technologies, USA), 1 μg/mL hydrocortisone (Sigma, St. Louis, USA), and 1% pen-strep antibiotic (Beyotime, China) in 6-well ultralow attachment plates (#3471, Corning, USA) (1 × 10^5^ cells/mL medium) for 7–10 days. Fresh complete mammocult medium was added every 3 days. After culture completed, spheres were collected and digested with 0.25% trypsin for about 6–8 minutes at 37°C. PBS with 2% FBS terminated digestion, and residual spheres not digested were removed by 40-μm filter. Cells were centrifuged with 1,200 rpm at 4°C for 5 minutes. Single cells were resuspended for subsequent experiments.

### Survival analysis with online database

GSE21653 data (cohort of basal breast cancer cases) [35] for OS and GSE25055 data (cohort of HER2-negative breast cancer cases) [36] for RFS analysis were from Gene Expression Omnibus (GEO) of National Center for Biotechnology Information (NCBI). Gene expression of CCL20 was bifurcated into high and low level at median. Available URL, https://www.ncbi.nlm.nih.gov/geo/query/acc.cgi. And results were obtained with online tool PROGgeneV2, for which the available URL is http://watson.compbio.iupui.edu/chirayu/proggene/database/?url=proggene.

### Immunofluorescence and confocal imaging

Cells of 0.1 million were seeded in chamber (154526, Thermo Scientific, USA) and cultured for 2 days. Cells were fixed with cold methanol, membrane perforated with 0.15% Triton X-100 (TB0198, Sangon Biotech), blocked with animal nonimmune serum (SP KIT-B, Maxvision), incubated with primary antibodies overnight at 4°C, and then incubated with secondary antibodies for 1 hour. Cell nucleus was stained with DAPI (P36931, Life Technologies, USA). Images were captured with confocal microscope (LSM 710, Zeiss, Germany) with 100× oil objective lens. The following antibodies were used for immune fluorescence: goat anti-rabbit IgG secondary antibody Alexa Fluor 546 (1:200, A11035, Invitrogen, USA).

### RNA-seq and data processing

Transcriptome sequencing was achieved by using the next-generation technology. After RNA extraction, the RNA integrality (RNA Quality Number) was first validated by Bioptic Qsep100, and the qualified RNA was used to establish an RNA library, which was checked by quality control with Bioptic Qsep100 before sequencing. When analyzing the expression levels of each gene in results of RNA-seq, the threshold of expression value was set to 0.1 greater than what was believed to be credible. When RNA-seq results were used for GSEA of each gene set, pathway of nominal *P* value less than 0.05 was considered significant.

### HPLC-MS

#### Standard curve and sample preparation

Solutions of different concentrations of docetaxel standard substance (purity ≥ 99%, D107319, Aladdin, China) (10, 50, 100, 200, 500, 1000, 2000 ng/mL) were prepared in 100 μL acetonitrile. Then, 10 μL of internal standard diphenhydramine (10 ng/ml, DPHM) (D129201, Aladdin, China) was added. Samples were vortex mixed for 3 minutes, stood for several minutes, and then were centrifuged at 4°C for 5 minutes at 14,000 rpm. Fifty μL of supernatant was for testing in HPLC-MS.

Cell precipitation was suspended with 500 μl acetonitrile, splintered by ultrasonic for 4 minutes, and centrifuged at 12,000 rpm for 5 minutes. Supernatants were placed in a new tube, dried at RT (CentriVap, LABCONCO, USA), and redissolved with 100 μl acetonitrile. Ten μL of internal standard DPHM (10 ng/ml) was added, vortex-mixed for 3 minutes, left standing for several minutes, and then centrifuged at 4°C for 5 minutes at 14,000 rpm. Fifty μL of supernatant was for testing in HPLC-MS.

#### Parameters of chromatography and mass spectrometry

HPLC-MS was carried out in ThermoFisher DIONEX UltiMate 3000/TSQ QUANTIVA (Thermo Fisher Scientific, USA). Chromatographic column used was Agilent ZORBAX Eclipse Plus C18 RRHD (Agilent, USA). In test method, ESI-MS and MRM of negative ion mode were used to monitor docetaxel and DPHM. Mobile phase: A: H_2_O+1% FA; B: ACN+1% FA at flow speed of 0.4 mL/min. Column temperature was 35°C, and 1 μL of sample was analyzed.

ACN, H_2_O, and FA used in sample preparation and HPLC-MS process were all LC-MS grade.

### ELISA

Serum and cell culture supernatants were used to perform ELISA to determine CCL20 levels, following manufacturer’s recommendations (CODE: ELH-MIP3a, RayBiotech, USA). CCL20 levels were calculated according to the standard curve after the subtraction of the negative control signal when measuring the level of CCL20 in the patient’s serum, which was called relative CCL20 level.

### Cytokine antibody array

Cell culture supernatants and mouse sera were collected and utilized to carry out human or mouse RayBio C-Series Cytokine Antibody Array (Cat# AAH-CYT-1000 or Cat# AAM-CYT-1000, RayBiotech, USA) per the manufacturer’s instruction. Briefly, the antibody array membranes were blocked with blocking buffer for 30 minutes at RT. Then, 1 mL of diluted or undiluted sample was pipetted into each well and incubated for 2 hours at RT. After washing process with wash buffer, biotinylated antibody cocktail was incubated on the membrane overnight at 4°C. Residual antibody cocktail was washed off, and HRP-Streptavidin incubation was conducted for 2 hours at RT. After the third wash, chemiluminescence detection was performed, and signal intensity was digitalized with ImageJ 1.49p software (NIH, USA).

### Plasmid/short hairpin RNA (shRNA) construction and virus infection

CCL20v1 and CCL20v2 were amplified from the reverse-transcribed cDNA from THP-1 cell line and cloned into pSIN (puromycin-resistant) vector (Addgene, USA), and the authenticity was verified by sequencing, respectively. Plasmid constructs expressing shRNAs were purchased from Sigma; the sense sequence was designed as follows in Table 1:

**Table 1:** 

shRNA target	Sequence (5′→3′)
shCCL20-2	CCGGCGAATCAGAAGCAGCAAGCAACTCGAGTTGCTTGCTGCTTCTGATTCGTTTTTG
shCCL20-5	CCGGATTGTGCGTCTCCTCAGTAAACTCGAGTTTACTGAGGAGACGCACAATTTTTTG
shp65-3	CCGGGCAGGCTATCAGTCAGCGCATCTCGAGATGCGCTGACTGATAGCCTGCTTTTT
shp65-5	CCGGCCTGAGGCTATAACTCGCCTACTCGAGTAGGCGAGTTATAGCCTCAGGTTTTT

Abbreviation: shRNA, short hairpin RNA.

A highly efficient lentiviral system was used to generate CCL20-overexpressing plasmid DNA and the shRNA plasmid DNA. The cell lines were infected with the lentiviruses, and the stable cell lines were established. The lentiviral transfection efficiency was over 90% in all cell lines.

### qRT-PCR

Total RNA was extracted with Trizol (9109, Takara), and the RNA concentration was measured with Nanodrop (Thermo Scientific, USA). One μg of RNA was reverse-transcribed to cDNA using ReverTra Ace qPCR RT Kit (FSQ-101, TOYOBO) and T100 Thermal Cycler (BIO-RAD). qRT-PCR was carried out to detect the expression level of related genes in this study using AceQ qPCR SYBR Green Master Mix (Q111, Vazyme), and it was performed using 7300Plus Real-Time PCR System (Applied Biosystems). RT-PCR data for mRNA are expressed relative to reference gene. The formula of relative expression value was as follows: 2 ^(−ΔCt). The gene-specific primers used are listed below in Table 2:

**Table 2:** 

Gene (*Homo sapiens*)	Primer pair	Sequence (5′→3′)
CCL20	Forward	TGCTGTACCAAGAGTTTGCTC
	Reverse	CGCACACAGACAACTTTTTCTTT
TBP	Forward	TGCACAGGAGCCAAGAGTGAA
	Reverse	CACATCACAGCTCCCCACCA
NANOG	Forward	AATACCTCAGCCTCCAGCAGATG
	Reverse	TGCGTCACACCATTGCTATTCTTC
OCT4	Forward	CTGGGTTGATCCTCGGACCT
	Reverse	CACAGAACTCATACGGCGGG
SOX2	Forward	GCACATGAACGGCTGGAGCAACG
	Reverse	TGCTGCGAGTAGGACATGCTGTAGG
p65	Forward	CTGCCGGGATGGCTTCTAT
	Reverse	CCGCTTCTTCACACACTGGAT
ABCB1	Forward	AAATTGGCTTGACAAGTTGTATATGG
	Reverse	CACCAGCATCATGAGAGGAAGTC

### Western blot

For sample preparation, when the cell culture reached 80% confluence, the cells were collected and split for 40 minutes on ice, the supernatant was denatured in 5x loading buffer containing SDS in boiling water for 10 minutes. We suggest not to boil the sample after lysis when preparing protein samples for ABCB1 determination by western blot. Next, 5% bovine serum albumin (BSA) dissolved in TBS containing 0.1% Tween-20 (TBST) was used for blocking and antibody dilution. The following antibodies and dilutions were used: actin (1:1,000, HC201, TransGen), CCL20 (1:1,000, AF360, R&D), p65 (1:1,000, 8242S), p-p65 (1:1,000, 3033S), p38 (1:1,000, 9212), p-p38 (1:1,000, 4631), p-PKCζ (1:1,000, 9378), ABCB1 (1:1,000, 13342S) (all from Cell Signaling Technology, USA), PKCζ (1:100, sc-17781, Santa Cruz), goat anti-mouse IgG-HRP (1:5,000, sc-2005, Santa Cruz), goat anti-rabbit IgG (1:5,000, sc-2004, Santa Cruz), donkey anti-goat IgG-HRP (1:2,000, SA00001-3, Proteintech). Western HRP Substrate (WBLUF0500, Millipore) was used to detect horseradish peroxidase–conjugated secondary antibodies.

### IHC

The tumor tissues of mice were fixed in formalin and processed for paraffin embedding. Sectioned samples were deparaffined in xylene and rehydrated in graded alcohol. Antigen retrieval was done according to the manufacture’s protocol (MVS-0100, Maxvision), and then the endogenous peroxidase was inactivated with 3% hydrogen peroxide methanol solution, blocked with animal nonimmune serum (SP KIT-B, Maxvision) and incubated with primary antibodies overnight at 4°C, and then incubated with secondary antibodies for 15 minutes. Slides were stained using the detection kit (DAB-0031, Maxvision); cell nucleus was stained with hematoxylin (ZLI-9610, ZSGB-BIO). The following antibodies and dilutions were used for IHC: CCL20 (1:50, Cat AF360, R&D), peroxidase-conjugated anti-goat secondary antibody (KIT 5107, Maxvision). Images were captured by trinocular microscope (OLYMPUS BX43, Japan). CCL20 expression was scored semiquantitatively by a manual histo-score (H-score) methodology based on staining intensity and percentage of positive tumor cells. Strongly staining scored 3, moderately staining scored 2, weakly staining scored 1, and negatively staining scored 0. The H-score of CCL20 expression is obtained by the formula 3 × percentage of strongly staining + 2 × percentage of moderately staining + percentage of weakly staining, giving a range of 0–300.

### MTT assay

Breast tumor cells (300–500 cells per well) were seeded in 96-well culture plates and cultured for 3, 5, or 7 days. MTT (M-2128, Sigma) was added to reach the final concentration of 0.5 mg/mL, and the cells were incubated at 37°C for 4 hours, and then the supernatant was removed, and 100 μl of DMSO was added; the optical density (OD) value was measured at 490 nm after gently shaking for 10 minutes.

### Invasion assay

Twenty thousand cells were seeded in the chambers with 8-μm pore size (0216, BD) coated with Matrigel (354234, Corning) and placed into 24-well culture plate. After 36 hours, cells in lower chambers were fixed and stained with 0.1% crystal violet, and the invaded cells were photographed by microscope to analyze the total numbers.

### Soft agar colony formation assay

Eight thousand cells per well were seeded in 2 X medium mixed with soft agar in 6-well culture plates and cultured in the incubator for approximately 4 weeks. Colonies were stained with 0.005% crystal violet, and then the number of colonies was analyzed.

### Statistical analysis

Bar graphs were generated with GraphPad Prism 7, and all values are reported as the mean ± SEM. A 2-way ANOVA was used for multiple comparisons. Unless otherwise indicated, comparisons between 2 groups were performed using an unpaired, 2-tailed *t* test. *P* values of less than 0.05 were considered statistically significant.

## Supporting information

S1 Fig CCL20 was one of the common elevated cytokines in the taxane-resistant breast cancer cells. (A-D) Cytokine antibody array was carried out with the 2-day FBS-free conditioned medium collected from SUM149 (A), SUM159 (C) after treatment with TAX (2 nM for SUM149, 10 nM for SUM159) or DOC (1 nM for SUM149, 5 nM for SUM159) for 7 days. Dots labeled with blue circle stand for CCL20. Heat maps (B and D) were clustered as described in Fig 1F. (E) List of cytokines that were up-regulated after DOC treatment in all tested 4 breast tumor cell lines (SUM149, SUM159, MDA-MB-231, and 4T1) and the representative patient serum (#26) during NAC as shown in the antibody arrays above (Fig 1, S1A and S1C Fig). The value shows the ratio of fold change over CTRL of the indicated cytokines. CCL20, C-C motif chemokine ligand 20; CTRL, control; DOC, docetaxel; FBS, fetal bovine serum; NAC, neoadjuvant chemotherapy; TAX, taxol.
(TIF)

S2 Fig CCL20 was induced in taxane-resistant TNBC cells in vitro. (A-B) SUM149, SUM159, and MDA-MB-231 cells were treated with TAX (2 nM for SUM149, 10 nM for SUM159, 13.46 nM for MDA-MB-231) or DOC (1 nM for SUM149, 5 nM for SUM159, 14.10 nM for MDA-MB-231) for 7 days. The mRNA levels of CCL20 in cells from different groups were measured by qRT-PCR (A). ***p < 0.001 versus CTRL by unpaired *t* test of triplicates. ELISA (B) was carried out with 2-day FBS-free conditioned medium after 7-day treatment, same as in (A). **p < 0.01, ***p < 0.001 versus CTRL by unpaired *t* test. Bar graphs are representative of duplicated experiments of ELISA and 3 repeats in each experiment. The data were shown as mean ± SEM. CCL20, C-C motif chemokine ligand 20; CTRL, control; DOC, docetaxel; ELISA, enzyme-linked immunosorbent assay; FBS, fetal bovine serum; qRT-PCR, quantitative real-time PCR; TAX, taxol; TNBC, triple-negative breast cancer.
(TIF)

S3 Fig The establishment of CCL20-knockdown and CCL20-overexpressing MDA-MB-231 cells and CCL20 promotion on breast cancer progression in SUM159 cells. (A-B) qRT-PCR (A) and western blot (B) were utilized to validate the knockdown of CCL20 in MDA-MB-231 cells. The immunoblotting bands were quantified, normalized with β-actin, and fold-changed to the first panel (similarly hereinafter). (C-D) qRT-PCR (C) and western blot (D) were utilized to validate the overexpression of CCL20 in MDA-MB-231 cells. (E-F) ELISA was conducted with supernatants of 2-day FBS-free medium after treatment for 3 days in SUM159 (E) and MDA-MB-231 (F). (G) MTT assay was conducted in vector control or CCL20-overexpressing SUM159 cells. (H-I) Matrigel invasion assay was carried out in vector control or CCL20-overexpressing SUM159 cells (H). Quantitative analysis of total invaded cells in (H) was shown as bar graphs (I). Scale bars: 200 μm. (J-K) Soft agar colony formation assay was performed with vector control or CCL20-overexpressing SUM159 cells. After 3–4 weeks, culture images of colony were captured (J), and the numbers of colonies were counted (K). (L) MTT assay was conducted in SUM159 cells in the presence or absence of rhCCL20 (10 ng/ml) or anti-CCL20 (200 ng/ml). (M) Matrigel invasion assay was carried out in SUM159 cells in presence or absence of rhCCL20 (10 ng/ml) or anti-CCL20 (200 ng/ml), and quantitative analysis of total invaded cells was shown as bar graphs. Data were shown as mean ± SEM and are representative of 3 individual experiments. *p < 0.05, **p < 0.01, ***p < 0.001 by unpaired *t* test of triplicates and multiple comparisons test of 2-way ANOVA (S3G and S3L). anti-CCL20, CCL20 neutralization antibody; CCL20, C-C motif chemokine ligand 20; ELISA, enzyme-linked immunosorbent assay; FBS, fetal bovine serum; MTT, 3-(4,5-dimethylthiazol-2-yl)-2,5 diphenyl tetrazolium bromide; qRT-PCR, quantitative real-time PCR; rhCCL20, recombinant human CCL20.
(TIF)

S4 Fig CCL20 enhanced the taxane resistance of TNBC through promoting ALDH^+^ breast cancer stem-like cells. (A) SUM149, SUM159, and MDA-MB-231 cells were treated with TAX (2 nM for SUM149, 10 nM for SUM159, 13.46 nM for MDA-MB-231) or DOC (1 nM for SUM149, 5 nM for SUM159, 14.10 nM for MDA-MB-231) for 7 days. Subsequently, the flow cytometry of Aldefluor Assay was performed to detect the ALDH^+^ population in these cells. The experiments were repeated 3 times, and the data were shown as mean ± SEM. (B) CCR6 level was determined by qRT-PCR in flow-sorted ALDH^+^ and ALDH^−^ cells. *p < 0.05 by unpaired *t* test. (C) ALDH^+^ and ALDH^−^ tumor cells were sorted from PDX (established by our group), and RNA-seq was conducted in these 2 subsets. CCR6 expression was shown. *p < 0.05 by unpaired *t* test. (D) The mRNA expression of stemness genes (NANOG, OCT4, and SOX2) was determined in mammospheres formed by vector or CCL20-overexpressing SUM159 cells by qRT-PCR. *p < 0.05 versus vector by unpaired *t* test. The data were shown as mean ± SEM. (E-F) Tumorsphere formation assay was conducted in vector or CCL20-overexpressing SUM159 cells. Representative images were shown (×100) (E), and bar graph showed the statistics of sphere numbers per field (×40) based on randomly selected 5 fields (F). ***p < 0.001 versus vector by unpaired *t* test. Data were shown as mean ± SEM. from 3 independent experiments. Scale bars: 400 μm. ALDH, aldehyde dehydrogenase; CCL20, C-C motif chemokine ligand 20; CCR6, C-C motif chemokine receptor type 6; DOC, docetaxel; FOV, field of view; PDX, patient-derived xenograft; qRT-PCR, quantitative real-time PCR; RNA-seq, RNA sequencing; TAX, taxol; TNBC, triple-negative breast cancer.
(TIF)

S5 Fig The promotion of NF-κB on CCL20-induced chemoresistance is mediated by PKCζ and p38 respectively. (A) Vector and CCL20v1-overexpressing SUM159 cells were cultured in the presence or absence of specific inhibitor of p65 NF-κB activation (CAPE, 5 μM) under FBS starvation conditions for 12 hours, and western blot was performed. (B) FBS-starved CCL20v1-overexpressing SUM159 cells were treated with PKCζ inhibitor (Go 6983, 5 μM) or p38 MAPK inhibitor (SB202190, 20 μM) for 12 hours and immunoblotted. (C) FBS-starved vector and CCL20v1-overexpressing MDA-MB-231 were treated (Go 6983, 5 μM; SB202190, 20 μM) for 12 hours and immunoblotted. (D-E) After DOC treatment (SUM159, 5 nM; MDA-MB-231, 14.10 nM) for 3 days in the presence or absence of PKCζ inhibitor (Go 6983, 5 μM) or p38 MAPK inhibitor (SB202190, 20 μM), chemoresistance index was determined in SUM159 (D) and MDA-MB-231 (E). (F) Single cells dissociated from SUM159 tumorspheres were treated with DOC (5 nM) for 24 hours and subjected to chemoresistance analysis. (G-H) Tumorsphere formation assay performed with SUM159 (G) and statistics (H). Scale bars: 400 μm. (I-K) Similar experiments conducted in MDA-MB-231 as in (F-H). (L) ALDH^+^ population was determined with Aldefluor assay in MDA-MB-231. Data are representative of at least 3 independent experiments and shown as mean ± SEM. **p < 0.01, ***p < 0.001 by unpaired *t* test of triplicates. ALDH, aldehyde dehydrogenase; CAPE, caffeic acid phenethyl ester; CCL20, C-C motif chemokine ligand 20; DOC, docetaxel; FBS, fetal bovine serum; Go, Gene Ontology; MAPK, mitogen-activated protein kinase; NF-κB, nuclear factor kappa B; n.s., not significant; PKCζ, protein kinase Cζ.
(TIF)

S6 Fig Up-regulation of ABCB1 by CCL20 promotes drug resistance through drug efflux. (A-B) Expression of ABCB1 was measured with qRT-PCR in flow cytometry–sorted ALDH^+^ and ALDH^−^ cells of SUM149 (A) and MDA-MB-231 (B). (C) Chromatogram of docetaxel and DPHM (internal standard) in the determination of docetaxel abundance through HPLC-MS. (D) SUM159 cells were treated with docetaxel (5 nM) for 3 days, and chemoresistance was determined. (E-F) SUM159 (E) and MDA-MB-231 (F) cells were treated with docetaxel (SUM159, 5 nM; MDA-MB-231, 14.10 nM) for 3 days in the presence or absence of CAPE (5 μM) and immunoblotted. Data shown are representative of at least 3 independent experiments and shown as mean ± SEM. *p < 0.05, **p < 0.01 by unpaired *t* test of triplicates. ABCB1, ATP-binding cassette subfamily B member 1; ALDH, aldehyde dehydrogenase; CAPE, caffeic acid phenethyl ester; CCL20, C-C motif chemokine ligand 20; DPHM, diphenhydramine; HPLC-MS, high-performance liquid chromatography–mass spectrometry; qRT-PCR, quantitative real-time PCR.
(TIF)

S7 Fig Effects of CCL20 and NF-κB blockade on CD24^−^CD44^+^ BCSCs in vivo. (A) In the experiments of Fig 7E, single cells from tumors were also determined for CD24^−^CD44^+^ population by flow cytometry. (B) Single cells from tumors in the experiments of Fig 7H were also determined for CD24^−^CD44^+^ population by flow cytometry. BCSC, breast cancer stem cell; CCL20, C-C motif chemokine ligand 20; NF-κB, nuclear factor kappa B.
(TIF)

S8 Fig CCL20 expression in non-TNBC breast cancer and other cancers. (A) CCL20 level was measured with qRT-PCR after TAX (10 nM) or DOC (5 nM) treatment for 3 days. (B) CCL20 level was determined with qRT-PCR in HCT-8 and TAX-resistant HCT-8/T colon cancer cells and in ovarian cancer cells of A2780 and TAX-resistant A2780/T. CCL20, C-C motif chemokine ligand 20; DOC, docetaxel; qRT-PCR, quantitative real-time PCR; TAX, taxol; TNBC, triple-negative breast cancer.
(TIF)

S1 Data Data used to generate the figures. (XLSX)

S2 Data RNA-seq data used in Fig 6. RNA-seq, RNA sequencing
(XLSX)

S1 Text Summary of the supporting information. (DOCX)

## Abbreviations

ABC: ATP-binding cassette
ABCB1: ATP-binding cassette subfamily B member 1
ALDH: aldehyde dehydrogenase
anti-CCL20: CCL20 neutralization antibody
BCSC: breast cancer stem cell
BSA: bovine serum albumin
CAPE: caffeic acid phenethyl ester
CCL20: C-C motif chemokine ligand 20
CCR6: C-C motif chemokine receptor type 6
CDX: cell line–derived xenograft
CR: complete response
DPHM: diphenhydramine
ELISA: enzyme-linked immunosorbent assay
ER: estrogen receptor
FACS: fluorescence-activated cell sorting
FOV: field of view
GEO: Gene Expression Omnibus
Go: Gene Ontology
GPCR: G protein–coupled receptor
GSEA: Gene Set Enrichment Analysis
HER2: human epidermal receptor 2
HPLC-MS: high-performance liquid chromatography–mass spectrometry
H-Score: histo-score
IHC: immunohistochemistry
IL-6: interleukin 6
MAPK: mitogen-activated protein kinase
MDR1: multidrug resistance 1
NAC: neoadjuvant chemotherapy
NCBI: National Center for Biotechnology Information
NF-κB: nuclear factor kappa B
OD: optical density
OS: overall survival
pCR: pathologic Complete Response
PDX: patient-derived xenograft
PKCζ: protein kinase Cζ
PR: progesterone receptor
qRT-PCR: quantitative real-time PCR
RA: rheumatoid arthritis
RFS: relapse-free survival
rhCCL20: recombinant human CCL20
RNA-seq: RNA sequencing
shCTRL: scramble control
shRNA: short hairpin RNA
TBST: TBS containing 0.1% Tween-20
TCGA: The Cancer Genome Atlas
TIS: therapeutic induced senescence
TNBC: triple-negative breast cancer
