# Supplementary figures and images for: CCL20 triggered by chemotherapy hinders the therapeutic efficacy of breast cancer
Source: PLoS Biol. 2018 Jul 27;16(7):e2005869. doi: 10.1371/journal.pbio.2005869 (PMC6082578; doi:10.1371/journal.pbio.2005869)

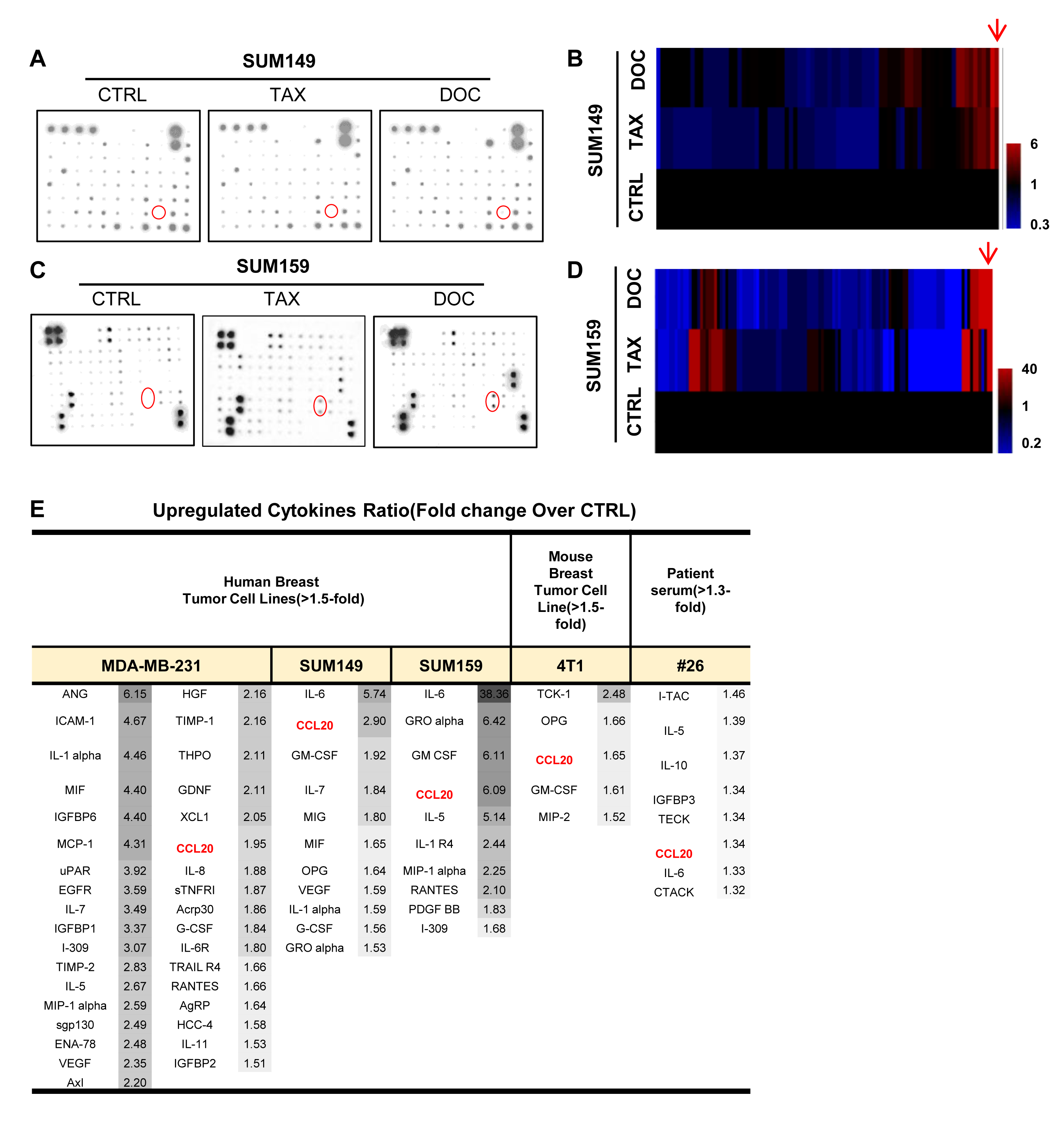

Supplement: S1 Fig — (A-D) Cytokine antibody array was carried out with the 2-day FBS-free conditioned medium collected from SUM149 (A), SUM159 (C) after treatment with TAX (2 nM for SUM149, 10 nM for SUM159) or DOC (1 nM for SUM149, 5 nM for SUM159) for 7 days. Dots labeled with blue circle stand for CCL20. Heat maps (B and D) were clustered as described in Fig 1F. (E) List of cytokines that were up-regulated after DOC treatment in all tested 4 breast tumor cell lines (SUM149, SUM159, MDA-MB-231, and 4T1) and the representative patient serum (#26) during NAC as shown in the antibody arrays above (Fig 1, S1A and S1C Fig). The value shows the ratio of fold change over CTRL of the indicated cytokines. CCL20, C-C motif chemokine ligand 20; CTRL, control; DOC, docetaxel; FBS, fetal bovine serum; NAC, neoadjuvant chemotherapy; TAX, taxol. (TIF) [file pbio.2005869.s001.tif]

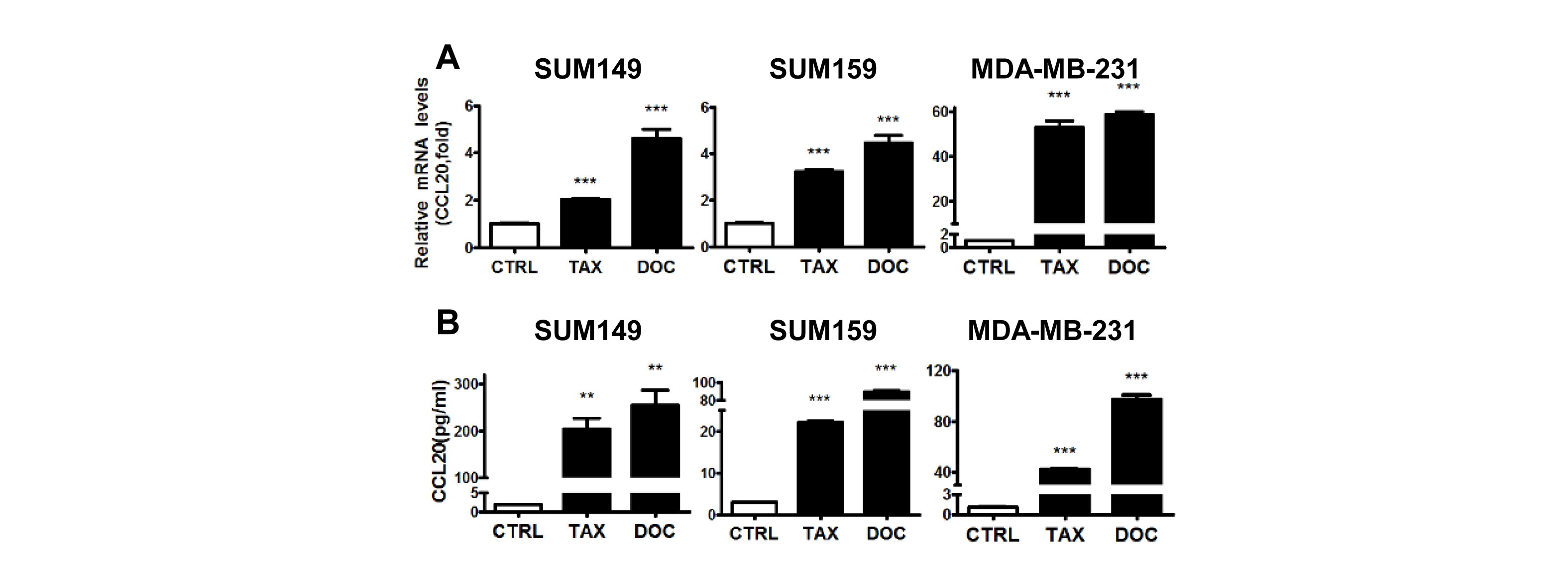

Supplement: S2 Fig — (A-B) SUM149, SUM159, and MDA-MB-231 cells were treated with TAX (2 nM for SUM149, 10 nM for SUM159, 13.46 nM for MDA-MB-231) or DOC (1 nM for SUM149, 5 nM for SUM159, 14.10 nM for MDA-MB-231) for 7 days. The mRNA levels of CCL20 in cells from different groups were measured by qRT-PCR (A). ***p < 0.001 versus CTRL by unpaired t test of triplicates. ELISA (B) was carried out with 2-day FBS-free conditioned medium after 7-day treatment, same as in (A). **p < 0.01, ***p < 0.001 versus CTRL by unpaired t test. Bar graphs are representative of duplicated experiments of ELISA and 3 repeats in each experiment. The data were shown as mean ± SEM. CCL20, C-C motif chemokine ligand 20; CTRL, control; DOC, docetaxel; ELISA, enzyme-linked immunosorbent assay; FBS, fetal bovine serum; qRT-PCR, quantitative real-time PCR; TAX, taxol; TNBC, triple-negative breast cancer. (TIF) [file pbio.2005869.s002.tif]

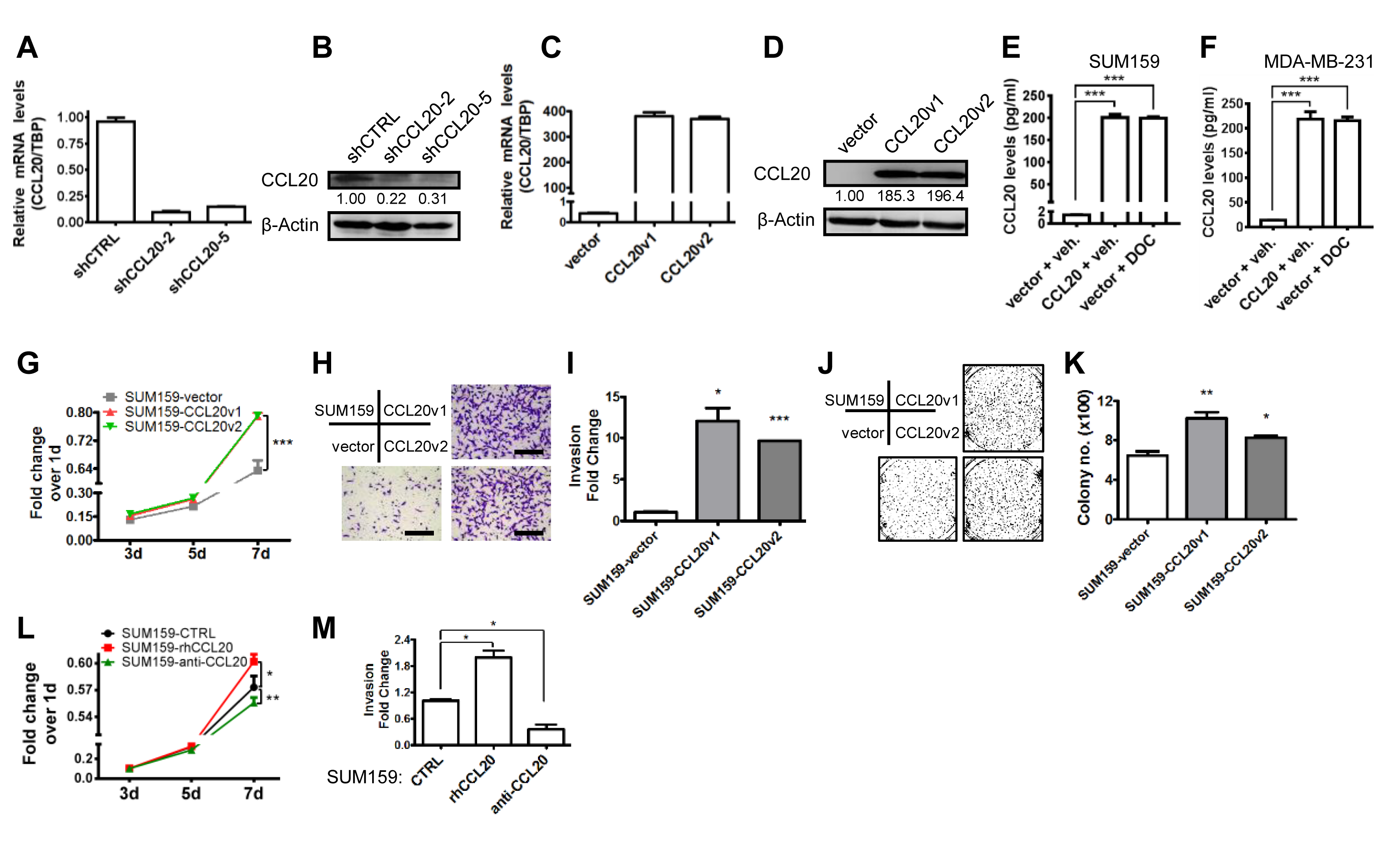

Supplement: S3 Fig — (A-B) qRT-PCR (A) and western blot (B) were utilized to validate the knockdown of CCL20 in MDA-MB-231 cells. The immunoblotting bands were quantified, normalized with β-actin, and fold-changed to the first panel (similarly hereinafter). (C-D) qRT-PCR (C) and western blot (D) were utilized to validate the overexpression of CCL20 in MDA-MB-231 cells. (E-F) ELISA was conducted with supernatants of 2-day FBS-free medium after treatment for 3 days in SUM159 (E) and MDA-MB-231 (F). (G) MTT assay was conducted in vector control or CCL20-overexpressing SUM159 cells. (H-I) Matrigel invasion assay was carried out in vector control or CCL20-overexpressing SUM159 cells (H). Quantitative analysis of total invaded cells in (H) was shown as bar graphs (I). Scale bars: 200 μm. (J-K) Soft agar colony formation assay was performed with vector control or CCL20-overexpressing SUM159 cells. After 3–4 weeks, culture images of colony were captured (J), and the numbers of colonies were counted (K). (L) MTT assay was conducted in SUM159 cells in the presence or absence of rhCCL20 (10 ng/ml) or anti-CCL20 (200 ng/ml). (M) Matrigel invasion assay was carried out in SUM159 cells in presence or absence of rhCCL20 (10 ng/ml) or anti-CCL20 (200 ng/ml), and quantitative analysis of total invaded cells was shown as bar graphs. Data were shown as mean ± SEM and are representative of 3 individual experiments. *p < 0.05, **p < 0.01, ***p < 0.001 by unpaired t test of triplicates and multiple comparisons test of 2-way ANOVA (S3G and S3L). anti-CCL20, CCL20 neutralization antibody; CCL20, C-C motif chemokine ligand 20; ELISA, enzyme-linked immunosorbent assay; FBS, fetal bovine serum; MTT, 3-(4,5-dimethylthiazol-2-yl)-2,5 diphenyl tetrazolium bromide; qRT-PCR, quantitative real-time PCR; rhCCL20, recombinant human CCL20. (TIF) [file pbio.2005869.s003.tif]

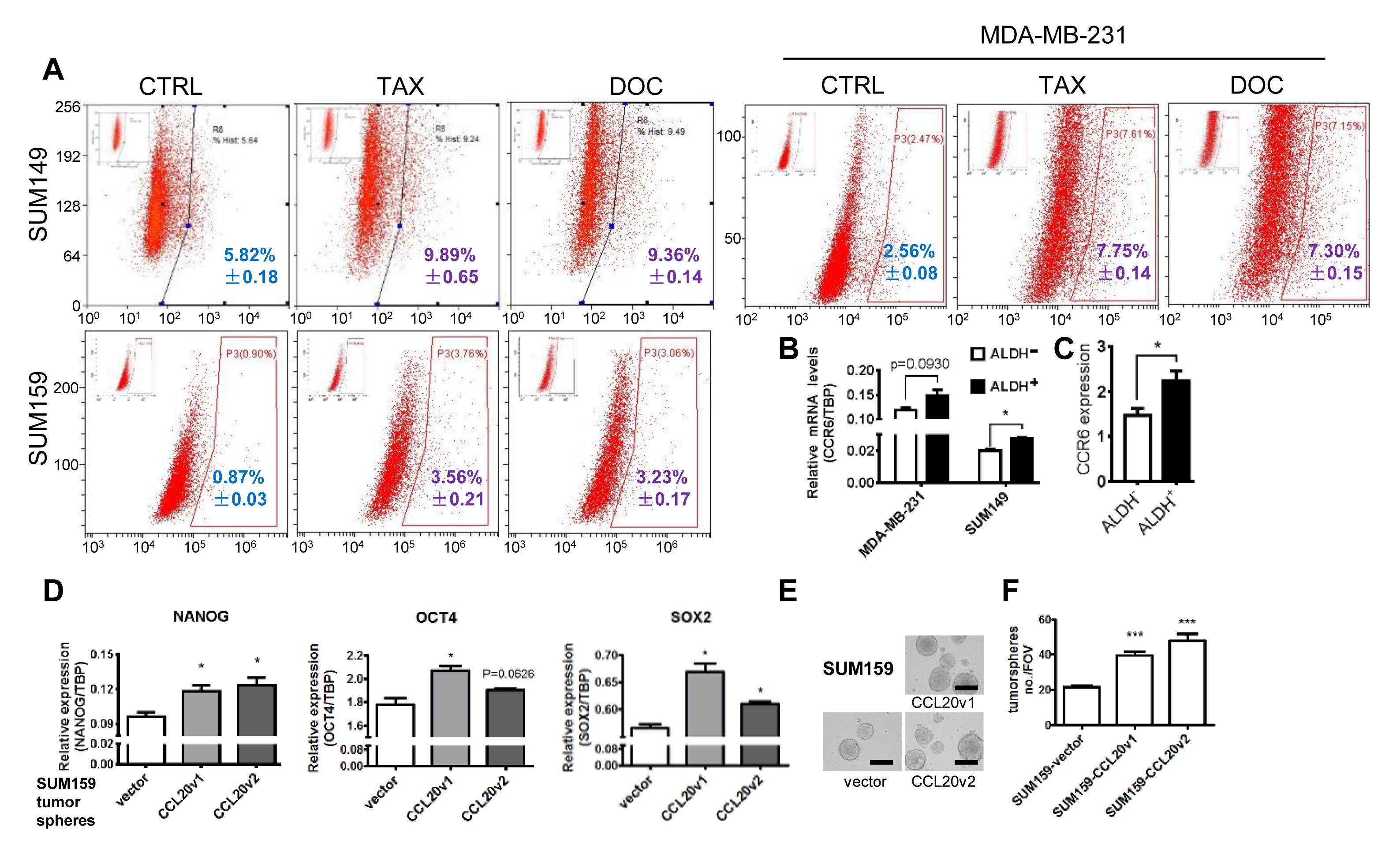

Supplement: S4 Fig — (A) SUM149, SUM159, and MDA-MB-231 cells were treated with TAX (2 nM for SUM149, 10 nM for SUM159, 13.46 nM for MDA-MB-231) or DOC (1 nM for SUM149, 5 nM for SUM159, 14.10 nM for MDA-MB-231) for 7 days. Subsequently, the flow cytometry of Aldefluor Assay was performed to detect the ALDH+ population in these cells. The experiments were repeated 3 times, and the data were shown as mean ± SEM. (B) CCR6 level was determined by qRT-PCR in flow-sorted ALDH+ and ALDH− cells. *p < 0.05 by unpaired t test. (C) ALDH+ and ALDH− tumor cells were sorted from PDX (established by our group), and RNA-seq was conducted in these 2 subsets. CCR6 expression was shown. *p < 0.05 by unpaired t test. (D) The mRNA expression of stemness genes (NANOG, OCT4, and SOX2) was determined in mammospheres formed by vector or CCL20-overexpressing SUM159 cells by qRT-PCR. *p < 0.05 versus vector by unpaired t test. The data were shown as mean ± SEM. (E-F) Tumorsphere formation assay was conducted in vector or CCL20-overexpressing SUM159 cells. Representative images were shown (×100) (E), and bar graph showed the statistics of sphere numbers per field (×40) based on randomly selected 5 fields (F). ***p < 0.001 versus vector by unpaired t test. Data were shown as mean ± SEM. from 3 independent experiments. Scale bars: 400 μm. ALDH, aldehyde dehydrogenase; CCL20, C-C motif chemokine ligand 20; CCR6, C-C motif chemokine receptor type 6; DOC, docetaxel; FOV, field of view; PDX, patient-derived xenograft; qRT-PCR, quantitative real-time PCR; RNA-seq, RNA sequencing; TAX, taxol; TNBC, triple-negative breast cancer. (TIF) [file pbio.2005869.s004.tif]

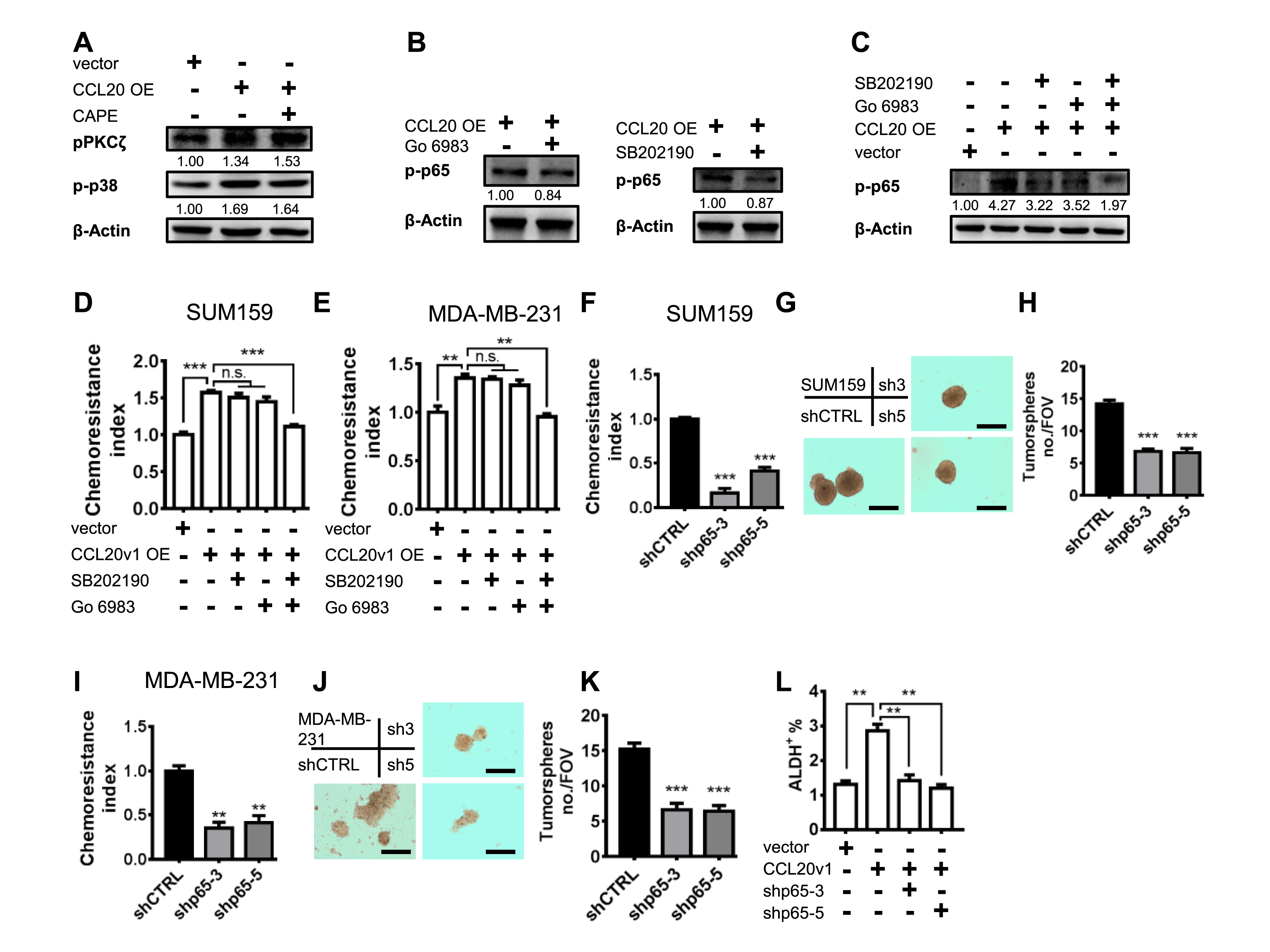

Supplement: S5 Fig — (A) Vector and CCL20v1-overexpressing SUM159 cells were cultured in the presence or absence of specific inhibitor of p65 NF-κB activation (CAPE, 5 μM) under FBS starvation conditions for 12 hours, and western blot was performed. (B) FBS-starved CCL20v1-overexpressing SUM159 cells were treated with PKCζ inhibitor (Go 6983, 5 μM) or p38 MAPK inhibitor (SB202190, 20 μM) for 12 hours and immunoblotted. (C) FBS-starved vector and CCL20v1-overexpressing MDA-MB-231 were treated (Go 6983, 5 μM; SB202190, 20 μM) for 12 hours and immunoblotted. (D-E) After DOC treatment (SUM159, 5 nM; MDA-MB-231, 14.10 nM) for 3 days in the presence or absence of PKCζ inhibitor (Go 6983, 5 μM) or p38 MAPK inhibitor (SB202190, 20 μM), chemoresistance index was determined in SUM159 (D) and MDA-MB-231 (E). (F) Single cells dissociated from SUM159 tumorspheres were treated with DOC (5 nM) for 24 hours and subjected to chemoresistance analysis. (G-H) Tumorsphere formation assay performed with SUM159 (G) and statistics (H). Scale bars: 400 μm. (I-K) Similar experiments conducted in MDA-MB-231 as in (F-H). (L) ALDH+ population was determined with Aldefluor assay in MDA-MB-231. Data are representative of at least 3 independent experiments and shown as mean ± SEM. **p < 0.01, ***p < 0.001 by unpaired t test of triplicates. ALDH, aldehyde dehydrogenase; CAPE, caffeic acid phenethyl ester; CCL20, C-C motif chemokine ligand 20; DOC, docetaxel; FBS, fetal bovine serum; Go, Gene Ontology; MAPK, mitogen-activated protein kinase; NF-κB, nuclear factor kappa B; n.s., not significant; PKCζ, protein kinase Cζ. (TIF) [file pbio.2005869.s005.tif]

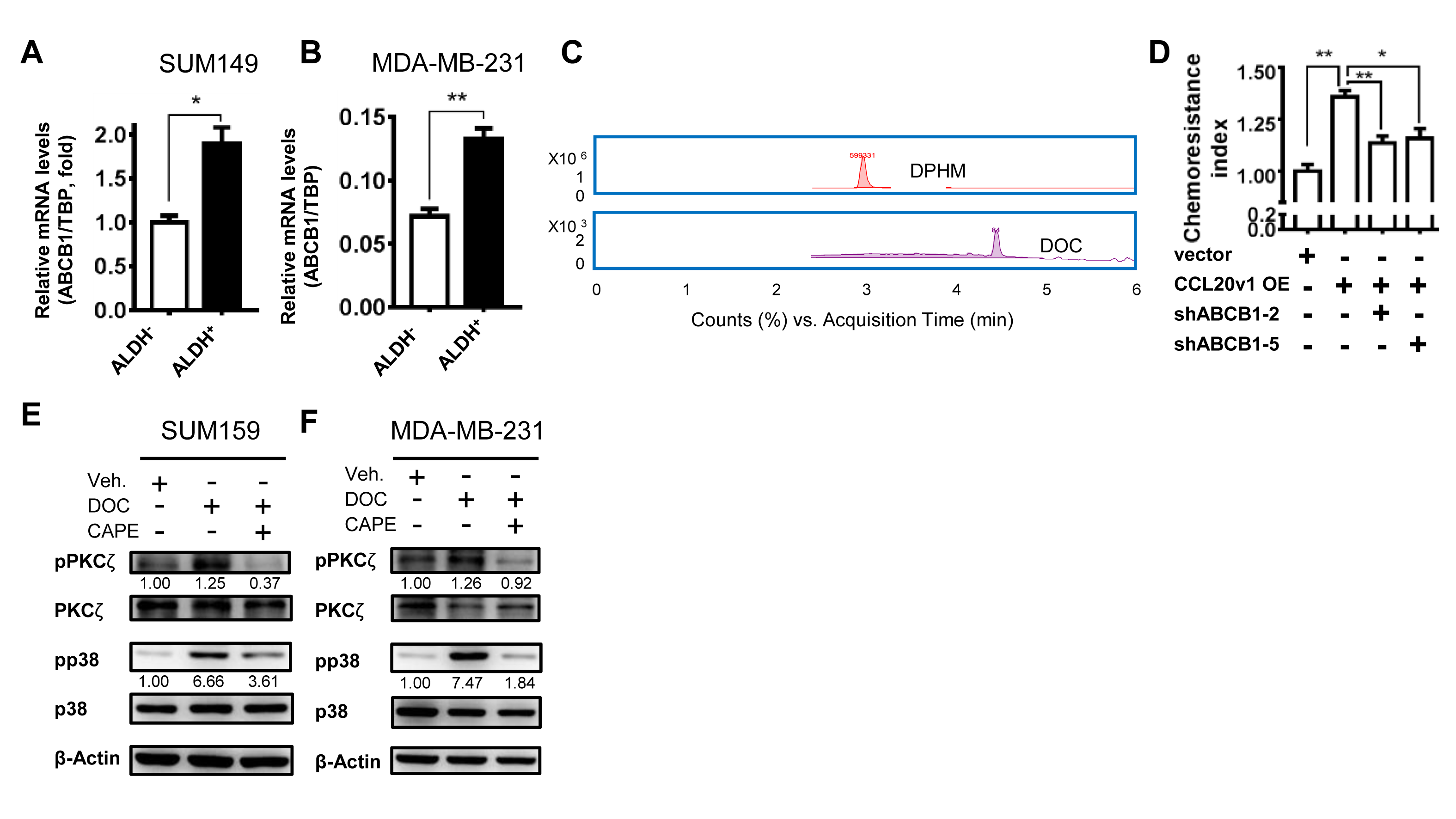

Supplement: S6 Fig — (A-B) Expression of ABCB1 was measured with qRT-PCR in flow cytometry–sorted ALDH+ and ALDH− cells of SUM149 (A) and MDA-MB-231 (B). (C) Chromatogram of docetaxel and DPHM (internal standard) in the determination of docetaxel abundance through HPLC-MS. (D) SUM159 cells were treated with docetaxel (5 nM) for 3 days, and chemoresistance was determined. (E-F) SUM159 (E) and MDA-MB-231 (F) cells were treated with docetaxel (SUM159, 5 nM; MDA-MB-231, 14.10 nM) for 3 days in the presence or absence of CAPE (5 μM) and immunoblotted. Data shown are representative of at least 3 independent experiments and shown as mean ± SEM. *p < 0.05, **p < 0.01 by unpaired t test of triplicates. ABCB1, ATP-binding cassette subfamily B member 1; ALDH, aldehyde dehydrogenase; CAPE, caffeic acid phenethyl ester; CCL20, C-C motif chemokine ligand 20; DPHM, diphenhydramine; HPLC-MS, high-performance liquid chromatography–mass spectrometry; qRT-PCR, quantitative real-time PCR. (TIF) [file pbio.2005869.s006.tif]

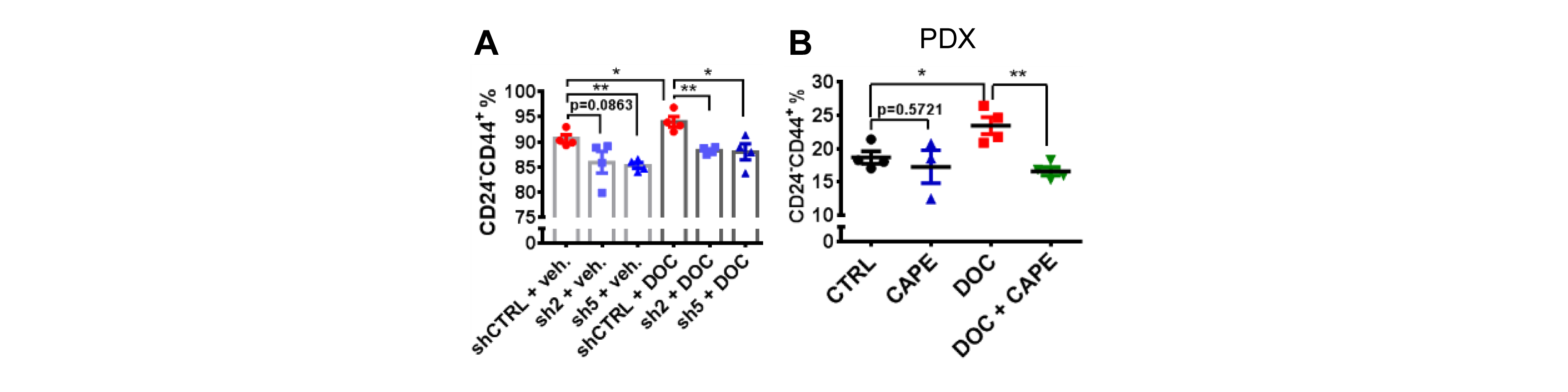

Supplement: S7 Fig — (A) In the experiments of Fig 7E, single cells from tumors were also determined for CD24−CD44+ population by flow cytometry. (B) Single cells from tumors in the experiments of Fig 7H were also determined for CD24−CD44+ population by flow cytometry. BCSC, breast cancer stem cell; CCL20, C-C motif chemokine ligand 20; NF-κB, nuclear factor kappa B. (TIF) [file pbio.2005869.s007.tif]

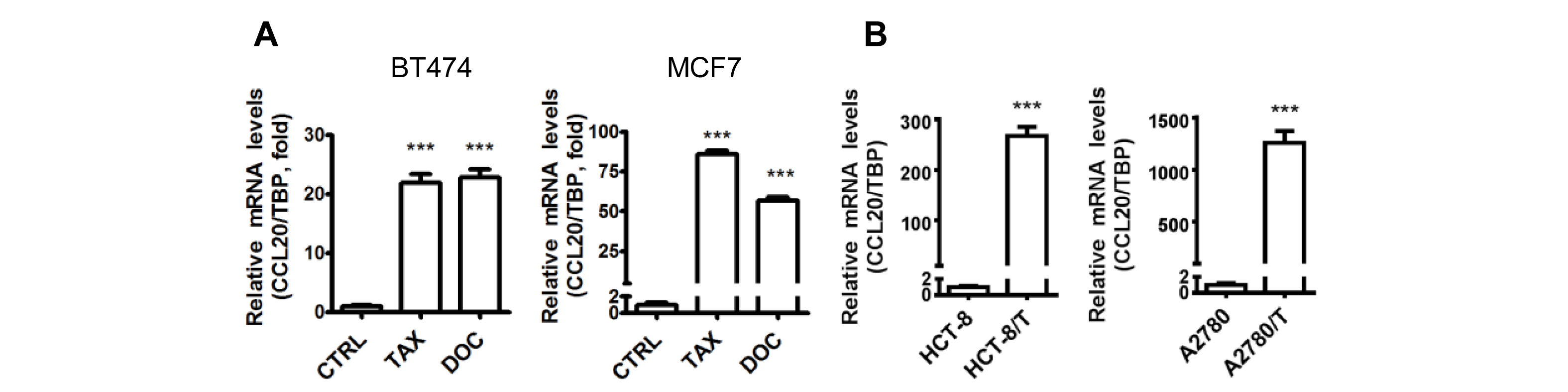

Supplement: S8 Fig — (A) CCL20 level was measured with qRT-PCR after TAX (10 nM) or DOC (5 nM) treatment for 3 days. (B) CCL20 level was determined with qRT-PCR in HCT-8 and TAX-resistant HCT-8/T colon cancer cells and in ovarian cancer cells of A2780 and TAX-resistant A2780/T. CCL20, C-C motif chemokine ligand 20; DOC, docetaxel; qRT-PCR, quantitative real-time PCR; TAX, taxol; TNBC, triple-negative breast cancer. (TIF) [file pbio.2005869.s008.tif]
