# Supplementary material for: CCL20 triggered by chemotherapy hinders the therapeutic efficacy of breast cancer
Source: PLoS Biol. 2018 Jul 27;16(7):e2005869. doi: 10.1371/journal.pbio.2005869 (PMC6082578; doi:10.1371/journal.pbio.2005869)
Supplement: S1 Text — (DOCX) [file pbio.2005869.s011.docx]

**Supporting information**

**Supplementary Figure legends**

**S1 Fig. CCL20 was one of the common elevated cytokines in the taxane-resistant breast cancer cells**

**(A-D)** Cytokine antibody array was carried out with the 2-day FBS-free conditioned medium collected from SUM149 (**A**), SUM159 (**C**) after treatment with taxol (TAX, 2nM for SUM149, 10nM for SUM159) or docetaxel (DOC, 1nM for SUM149, 5nM for SUM159) for 7 days. Dots labeled with blue circle stands for CCL20. Heatmaps (**B** and **D**) were clustered as described in Figure 1F. **(E)** List of cytokines which were upregulated after docetaxel treatment in all tested four breast tumor cell lines (SUM149, SUM159, MDA-MB-231 and 4T1) and the representative patient serum (#26) during NAC as shown in the antibody arrays as shown above. The value shows the ratio of fold change over CTRL of the indicated cytokines.

**S2 Fig. CCL20 was induced in taxane-resistant TNBC cells in vitro**

**(A-B)** SUM149, SUM159 and MDA-MB-231 cells were treated with TAX (2nM for SUM149, 10nM for SUM159, 13.46nM for MDA-MB-231) or DOC (1nM for SUM149, 5nM for SUM159, 14.10nM for MDA-MB-231) for 7 days. The mRNA levels of CCL20 in cells from different groups were measured by qRT-PCR (**A**). ***p<0.001 versus CTRL by Unpaired t test of triplicates. ELISA (**B**) was carried out with 2-day FBS-free conditioned medium after 7-day treatment same as in (**A**). **p<0.01, ***p<0.001 versus CTRL by Unpaired t test. Bar graphs are representative of duplicated experiments of ELISA and three repeats in each experiment. The data were shown as mean ± SEM.

**S3 Fig. The establishment of CCL20-knockdown and -overexpressing MDA-MB-231 cells and CCL20 promotion on breast cancer progression in SUM159 cells**

**(A-B)** qRT-PCR (**A**) and western blot (**B**) were utilized to validate the knockdown of CCL20 in MDA-MB-231 cells. The immunoblotting bands were quantified, normalized with β-actin and fold-changed to the first panel (similarly hereinafter). **(C-D)** qRT-PCR (**C**) and western blot (**D**) were utilized to validate the overexpression of CCL20 in MDA-MB-231 cells. (**E-F**) ELISA was conducted with supernatants of two-day FBS-free medium after treatment for 3 days in SUM159 (E) and MDA-MB-231 (F). **(G)** MTT assay was conducted in vector control or CCL20-overexpressing SUM159 cells. **(H-I)** Matrigel invasion assay was carried out in vector control or CCL20-overexpressing SUM159 cells (**H**). Quantitative analysis of total invaded cells in (**H**) was shown as bar graphs (**I**). Scale bars: 200μm. **(J-K)** Soft agar colony formation assay was performed with vector control or CCL20-overexpressing SUM159 cells. After 3-4 week culture images of colony were captured (**J**) and the numbers of colonies were counted (**K**). **(L)** MTT assay was conducted in SUM159 cells in the presence or absence of rhCCL20 (10ng/ml) or anti-CCL20 (200ng/ml). **(M)** Matrigel invasion assay was carried out in SUM159 cells in presence or absence of rhCCL20 (10ng/ml) or anti-CCL20 (200ng/ml) and quantitative analysis of total invaded cells was shown as bar graphs. Data were shown as mean ± SEM. and are the representative of three individual experiments. *p<0.05, **p<0.01, ***p<0.001 by Unpaired t test of triplicates and multiple comparisons test of Two-way ANOVA (**S3G** and **S3L**).

**S4 Fig. CCL20 enhanced the taxane-resistance of TNBC through promoting ALDH^+^ breast cancer stem-like cells**

**(A)** SUM149, SUM159 and MDA-MB-231 cells were treated with TAX (2nM for SUM149, 10nM for SUM159, 13.46nM for MDA-MB-231) or DOC (1nM for SUM149, 5nM for SUM159, 14.10nM for MDA-MB-231) for 7 days. Subsequently, the flow cytometry of Aldefluor Assay was performed to detect the ALDH^+^ population in these cells. The experiments were repeated three times and the data were shown as mean ± SEM. **(B)** CCR6 level was determined by qRT-PCR in flow-sorted ALDH^+^ and ALDH^-^ cells. *p<0.05 by Unpaired t test. **(C)** ALDH^+^ and ALDH^-^ tumor cells were sorted from patient-derived xenograft (PDX, established by our group) and RNA-seq was conducted in these two subsets. CCR6 expression was shown. *p<0.05 by Unpaired t test. **(D)** The mRNA expression of stemness genes (NANOG, OCT4 and SOX2) were determined in mammospheres formed by vector or CCL20-overexpressing SUM159 cells by qRT-PCR. *p<0.05 vs. vector by Unpaired t test. The data were shown as mean ± SEM. **(E-F)** Tumorsphere formation assay was conducted in vector or CCL20-overexpressing SUM159 cells. Representative images were shown (×100) (**E**) and bar graph showed the statistics of sphere numbers per field (×40) based on randomly selected five fields (**F**). ***p<0.001 vs. vector by Unpaired t test. FOV, Field of View. Data were shown as mean ± SEM. from 3 independent experiments. Scale bars: 400μm.

**S5 Fig. The promotion of NF-κB on CCL20-induced chemoresistance is mediated by PKCζ and p38 respectively.**

(**A**) Vector and CCL20v1-overexpressing SUM159 cells were cultured in the presence or absence of specific inhibitor of p65 NF-κB activation (CAPE, 5uM) under FBS starvation conditions for 12h and western blot was performed. (**B**) FBS-starved CCL20v1-overexpressing SUM159 cells were treated with PKCζ inhibitor (Go 6983, 5uM) or p38 MAPK inhibitor (SB202190, 20uM) for 12h and immunoblotted. (**C**) FBS-starved vector and CCL20v1-overexpressing MDA-MB-231 were treated (Go 6983, 5uM; SB202190, 20uM) for 12h and immunoblotted. (**D-E**) After docetaxel treatment (SUM159, 5nM; MDA-MB-231, 14.10nM) for 3 days in the presence or absence of PKCζ inhibitor (Go 6983, 5uM) or p38 MAPK inhibitor (SB202190, 20uM) chemoresistance index was determined in SUM159 (D) and MDA-MB-231 (E). (**F**) Single cells dissociated from SUM159 tumorspheres were treated with DOC (5nM) for 24h and subjected to chemoresistance analysis. (**G-H**) Tumorsphere formation assay performed with SUM159 (G) and statistics (H). Scale bars: 400μm. (**I-K**) Similar experiments conducted in MDA-MB-231 as in (F-H). (**L**) ALDH^+^ population was determined with Aldefluor assay in MDA-MB-231. Data are representative of at least three independent experiments and shown as mean ± SEM. **p<0.01, ***p<0.001 by Unpaired t test of triplicates; n.s., not significant.

**S6 Fig. Upregulation of ABCB1 by CCL20 promotes drug resistance through drug efflux.**

(**A-B**) Expression of ABCB1 was measured with qRT-PCR in flow cytometry-sorted ALDH^+^ and ALDH^-^ cells of SUM149 (A) and MDA-MB-231 (B). (**C**) Chromatogram of docetaxel and diphenhydramine (DPHM, internal standard) in the determination of docetaxel abundance through HPLC-MS. (**D**) SUM159 cells were treated with docetaxel (5nM) for 3 days and chemoresistance was determined. (**E-F**) SUM159 (E) and MDA-MB-231 (F) cells were treated with docetaxel (SUM159, 5nM; MDA-MB-231, 14.10nM) for 3 days in the presence or absence of CAPE (5uM) and immunoblotted. Data shown is the representative of at least three independent experiments and shown as mean ± SEM. *p<0.05, **p<0.01 by Unpaired t test of triplicates.

**S7 Fig. Effects of CCL20 and NF-κB blockade on CD24^-^CD44^+^ BCSCs *in vivo***

(**A**) In the experiments of Figure 7E, single cells from tumors were also determined for CD24^-^CD44^+^ population by flow cytometry. (**B**) Single cells from tumors in the experiments of Figure 7H were also determined for CD24^-^CD44^+^ population by flow cytometry.

**S8 Fig. CCL20 expression in non-TNBC breast cancer and other cancers**

(A) CCL20 level was measured with qRT-PCR after TAX (10nM) or DOC (5nM) treatment for 3 days. (B) CCL20 level was determined with qRT-PCR in HCT-8 and taxol-resistant HCT-8/T colon cancer cells, and in ovarian cancer cells of A2780 and taxol-resistant A2780/T.

**S1 Data. Data used to generate the figures.**

**S2 Data. RNA-seq data used in Figure 6.**

**S1 Text. Summary of the supporting information.**
